# Sculpting the Bacterial *O*-Glycoproteome: Functional Analyses of Orthologous Oligosaccharyltransferases with Diverse Targeting Specificities

**DOI:** 10.1128/mbio.03797-21

**Published:** 2022-04-26

**Authors:** Chris Hadjineophytou, Jan Haug Anonsen, Tina Svingerud, Tatum D. Mortimer, Yonatan H. Grad, Nichollas E. Scott, Michael Koomey

**Affiliations:** a Department of Biosciences, Section for Genetics and Evolutionary Biology, University of Oslo, Oslo, Norway; b Department of Immunology and Infectious Diseases, Harvard T. H. Chan School of Public Health, Boston, Massachusetts, USA; c Division of Infectious Diseases, Brigham and Women’s Hospital and Harvard Medical School, Boston, Massachusetts, USA; d Department of Microbiology and Immunology, University of Melbourne at the Peter Doherty Institute for Infection and Immunity, Melbourne, Australia; e Department of Biosciences, Centre for Ecological and Evolutionary Synthesis, University of Oslo, Oslo, Norway; University of Michigan—Ann Arbor

**Keywords:** *Neisseria*, evolution, glycoproteins, oligosaccharides, pili

## Abstract

Protein glycosylation systems are widely recognized in bacteria, including members of the genus *Neisseria*. In most bacterial species, the molecular mechanisms and evolutionary contexts underpinning target protein selection and the glycan repertoire remain poorly understood. Broad-spectrum *O*-linked protein glycosylation occurs in all human-associated species groups within the genus *Neisseria*, but knowledge of their individual glycoprotein repertoires is limited. Interestingly, PilE, the pilin subunit of the type IV pilus (Tfp) colonization factor, is glycosylated in Neisseria gonorrhoeae and Neisseria meningitidis but not in the deeply branching species *N. elongata* subsp. *glycolytica*. To examine this in more detail, we assessed PilE glycosylation status across the genus and found that PilEs of commensal clade species are not modified by the gonococcal PglO oligosaccharyltransferase. Experiments using PglO oligosaccharyltransferases from across the genus expressed in N. gonorrhoeae showed that although all were capable of broad-spectrum protein glycosylation, those from a deep-branching group of commensals were unable to support resident PilE glycosylation. Further glycoproteomic analyses of these strains using immunoblotting and mass spectrometry revealed other proteins differentially targeted by otherwise remarkably similar oligosaccharyltransferases. Finally, we generated *pglO* allelic chimeras that begin to localize PglO protein domains associated with unique substrate targeting activities. These findings reveal previously unappreciated differences within the protein glycosylation systems of highly related bacterial species. We propose that the natural diversity manifest in the neisserial protein substrates and oligosaccharyltransferases has significant potential to inform the structure-function relationships operating in these and related bacterial protein glycosylation systems.

## INTRODUCTION

Glycosylation is an important element of biological systems across all domains of life through coordinated posttranslational modification of large sets of proteins. Although significant progress has been made in defining the basic enzymatic pathways of many such systems, the processes by which particular proteins are targeted and specific sites within proteins are selected remain incompletely understood. The latter situations persist despite the increasing availability of high-resolution structures for glycan transferases and the identification of domains and motifs inherent to protein target substrates. These gaps in knowledge limit efforts to understand the molecular and evolutionary processes that ultimately define and shape glycoproteomes.

In Gram-negative organisms, the process of broad-spectrum (or general) protein glycosylation is primarily localized to the periplasm and catalyzed by oligosaccharyltransferases (OTases) that are members of the GT-C superfamily (1). These OTases share structural features of multiple, transmembrane helices and the utilization of lipid-linked glycan donors. These include the asparagine-targeting OTases exemplified by Campylobacter jejuni PglB (2), as well as a family of OTases that target serine sites and have been documented within *Neis*seria (3, 4), Acinetobacter (5), *Ralstonia* (6), *Francisella* (7), *Burkholderia* (8), *Vibrio* (8), and Mycobacterium (9) species. The latter class of transferases are termed *O*-OTases based on their *O*-linked glycosylating activities and recognize serine attachment sites within low-complexity regions (LCRs) rich in alanine and proline. Campylobacter PglBs and *O*-OTases are also members of the shape, elongation, division, and sporulation (SEDS) protein family of transferases involved in cell wall biogenesis and remodeling and modification of surface glycoconjugates (2). While molecular structures have been solved for orthologous *N*-OTases from bacteria, archaea, and eukaryotes and SEDS proteins (9–13), little is known regarding the structure-function relationships of *O*-OTases.

A unique subset of *O*-OTases found in isolates of Pseudomonas aeruginosa and Acinetobacter species solely glycosylate subunits of type IV pili (Tfp) or pilin-like molecules (14–16). In the species Neisseria gonorrhoeae, Neisseria meningitidis, Ralstonia solanacearum, and Francisella tularensis, Tfp pilins are among the protein targets of the broad-spectrum *O*-OTases (3, 6, 7, 17). In contrast, in Neisseria elongata subsp. *glycolytica* (here referred to as *N. elongata*), the broad-spectrum *O*-OTase does not glycosylate its Tfp subunit protein (18). Therefore, a variety of strategies have emerged regarding the adaptability of *O*-OTases to target specific sets of proteins in general and Tfp pilin proteins in particular.

Members of the genus *Neisseria* colonizing mucosal sites of humans include the important pathogens N. gonorrhoeae and N. meningitidis, as well as additional species groups that are commensal inhabitants. Given their genetic relatedness, common ancestry, and host restriction, these species groups encompass a unique model system in which to study the evolution and ecology of host-microbe interactions. Based on these observations, we set out to examine species-level relationships between PilE glycosylation and *O*-OTase targeting activities when expressed in gonococci as a defined reference background. The findings strongly suggest that glycoprotein repertoires across the genus are shaped by distinct targeting activities of *O*-OTases together with intrinsic structural features of substrate proteins. We propose that standing genetic variation within the genus *Neisseria* provides unique resources to understand the structure-function relationships of protein targeting glycosyltransferases and their protein substrates.

## RESULTS

### Identification of candidate Tfp subunit genes and validation by complementation in N. gonorrhoeae.

**FIG 1:**
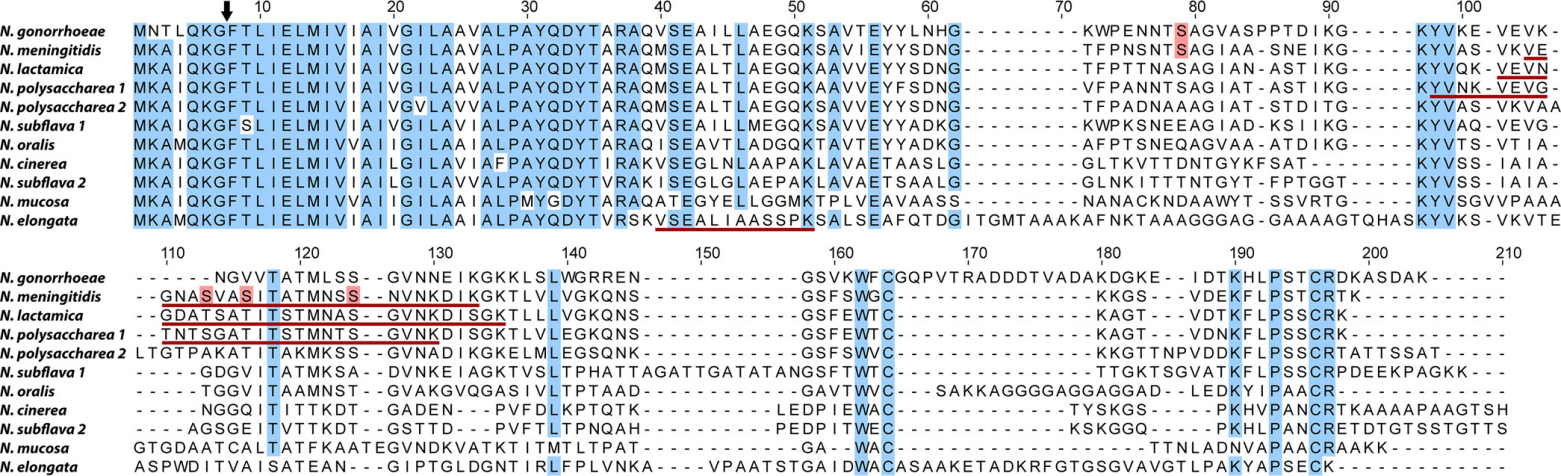
Structural alignment and features of defined and candidate neisserial PilE pilin subunits. The cleavage site for processing to mature pilin is marked with an arrow at the conserved Gly_−1_Phe_+1_ junction residues. Residues present in at least 10 of the 11 PilE orthologs are highlighted in blue, and established sites of glycan attachment are highlighted in red. Glycopeptides identified in this study are underlined in red (see also Fig. S3). The strains used to generate these data are listed in Table S1A.

**FIG 2:**
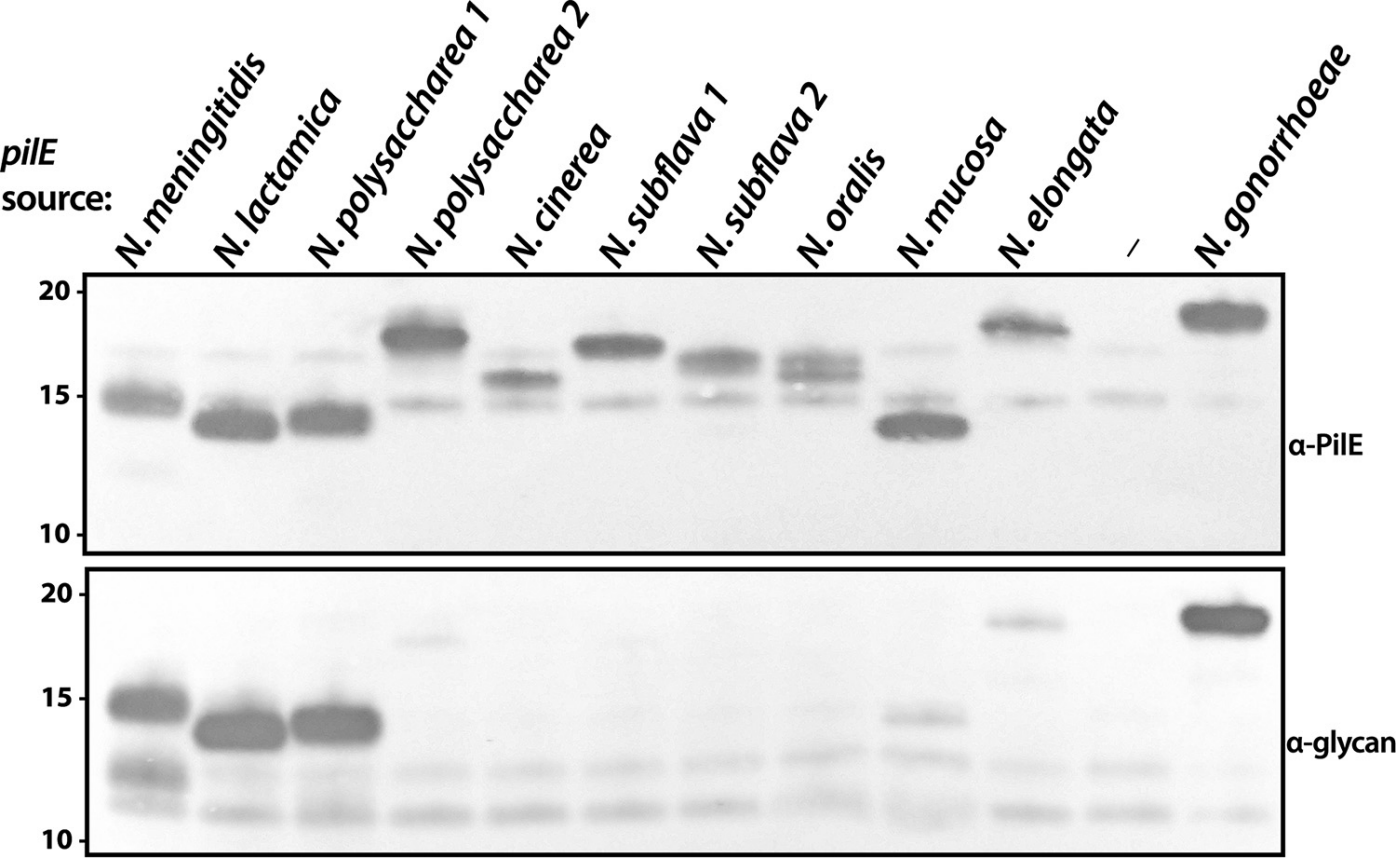
Neisserial Tfp pilins vary in glycosylation susceptibility when expressed in N. gonorrhoeae. Immunoblots were performed using equal amounts of bacterial whole-cell lysates. (Top panel) Samples probed with a polyclonal antiserum raised against purified N. gonorrhoeae pili from strain MS11. (Bottom panel) The same samples as in the top panel probed with the glycan-specific monoclonal antibody. The strains used are (from left to right) CH24, CH16, CH12, CH78, CH8, CH20, CH28, CH32, CH36, CH4, KS122, and CH114.

While studies have identified putative *pilE* genes in other neisserial species groups (19, 20), only those from N. gonorrhoeae, N. meningitidis, and *N. elongata* have been conclusively validated as such. We first aimed to identify orthologous *pilE* candidates within the genomes of other species available in the PubMLST database using BLASTp analyses and BLASTn with PilE sequences from N. gonorrhoeae and *N. elongata* as queries (Fig. 1; see Fig. S1A in the supplemental material). Candidate orthologues from each type strain were expressed in N. gonorrhoeae and assessed for their ability to complement Tfp-associated phenotypes. These included those from two type strains identified as isolates of Neisseria polysaccharea, but for which further analyses had defined as being members of two unique clusters (designated here as N. polysaccharea 1 and 2) and two strains of Neisseria subflava, one of which was previously identified as Neisseria flavescens (designated here as *N. subflava* 1 and 2, respectively) (21, 22). Immunoblotting using an antiserum recognizing a conserved epitope within Tfp pilins confirmed expression of each of the candidate PilEs (Fig. 2, top panel). Competence for natural transformation was assessed as Tfp expression is essential for this phenotype. All alleles tested supported high levels of transformability, being over 1,000-fold above the *pilE* negative-control background (Fig. 1B). Although there were differences in the abundance of Tfp-like appendages seen by transmission electron microscopy (TEM) (Fig. S1C), peptides corresponding to each of the open reading frames (ORFs) were detected in enriched shear extract fractions by mass spectrometry (unpublished data).

10.1128/mbio.03797-21.1 FIG S1 (A) Neighbor-joining phylogenetic tree of candidate neisserial *pilE* genes. The tree was based on a MAFFT alignment (1) of 93 unique putative *pilE* sequences derived from PubMLST and visualized with Interactive Tree of Life (2). The alleles are color coded for each species and presented with their PubMLST ID number followed by their abbreviated species name and strain. Two PilE alleles from N. gonorrhoeae and N. meningitidis are set as reference sequences. Candidate *pilE* genes expressed in N. gonorrhoeae for glycosylation experiments are marked with an asterisk (*) and are listed in Table S1. Consensus support values of 1,000 bootstrap replicates are shown for branches with ≥80% confidence. (B) Complementation of transformation competence by candidate PilEs confirms their functionality as Tfp-forming pilin subunits. Transformation frequencies are plotted as resistant CFU divided by total CFU and shown as the mean from three independent experiments (+ standard deviation [SD]). ND, no transformants detected. The strains used are (from left to right) CH114, CH24, CH16, CH12, CH78, CH8, CH20, CH28, CH32, CH36, CH4, KS122, and CH114. (C) Piliation and pilin glycosylation of N. gonorrhoeae strains expressing exogenous PilE proteins analyzed by immunogold TEM. Samples of intact cells placed directly on grids were negatively stained, and glycan epitopes were detected using the MAb npg1. In strains where glycan labeling was observed, experiments using isogenic strains defective in glycan synthesis (carrying a *pglC* null mutation) were assessed as negative controls. Download FIG S1, TIF file, 19.1 MB. Copyright © 2022 Hadjineophytou et al. 2022 Hadjineophytou et al. https://creativecommons.org/licenses/by/4.0/ This content is distributed under the terms of the Creative Commons Attribution 4.0 International license.

10.1128/mbio.03797-21.3 FIG S3 Identification of glycopeptides derived from trypsin-treated PilE pilins expressed in N. gonorrhoeae. The reporter ions of glycans representing diNAcBac-AcGal, diNAcBac-Gal, and diNAcBAc can be detected in the low-mass area at *m*/*z* values of 433.181, 391.170, and 229.118, respectively. (A) MS2 spectra of the glycopeptide VEGNASVASITATMNSSNVNKDIK derived from N. meningitidis FAM18 (strain CH22) carrying two copies of diNAcBac-AcGal (triply charged precursor ion at *m*/*z* 1,105.526), two copies of diNAcBac-Gal (triply charged precursor ion at *m*/*z* 1,077.517), a single diNAcBac-AcGal (triply charged precursor ion at *m*/*z* 959.820) eluting as two distinct peptide species in the chromatogram, and a single diNAcBac-Gal (triply charged precursor ion at *m*/*z* 947.796). (B) MS2 spectra of the glycopeptide YVNKVEVGTNTSGATITSTMNTSGVNK derived from PilE from N. polysaccharea ATCC 43768 (strain CH10) carrying a single diNAcBac (triply charged precursor ion at *m*/*z* 1,001.831), a single diNAcBac-Gal (triply charged precursor ion at *m*/*z* 792.138), or a single diNAcBac-Gal (triply charged precursor ion at *m*/*z* 802.391). (C) MS2 spectra of the glycopeptide VEVNGDATSATITSTMNASGVNKDISGK derived from PilE from N. lactamica ATCC 23970 (strain CH14) carrying a single diNAcBac-Gal (triply charged precursor ion at *m*/*z* 1,053.841). (D) MS2 spectra of the glycopeptide VSEALIAASSPK derived from PilE from *N. elongata* ATCC 29315 (strain CH2) carrying a single diNAcBac-AcGal (doubly charged precursor ion at *m*/*z* 803.419), a diNAcBac-Gal (doubly charged precursor ion at *m*/*z* 802.391), or a diNAcBac (doubly charged precursor ion at *m*/*z* 701.385). Download FIG S3, TIF file, 0.5 MB. Copyright © 2022 Hadjineophytou et al. 2022 Hadjineophytou et al. https://creativecommons.org/licenses/by/4.0/ This content is distributed under the terms of the Creative Commons Attribution 4.0 International license.

10.1128/mbio.03797-21.10 TABLE S1 (A) Strains and plasmids. (B) PCR oligonucleotide primers. (C) List of isolates used for genus-wide phylogenetic analyses in *Neisseria.* Download Table S1, DOCX file, 0.1 MB. Copyright © 2022 Hadjineophytou et al. 2022 Hadjineophytou et al. https://creativecommons.org/licenses/by/4.0/ This content is distributed under the terms of the Creative Commons Attribution 4.0 International license.

### *Neisseria* Tfp pilins vary in glycosylation susceptibility when expressed in N. gonorrhoeae.

Having confirmed that the genes identified encode Tfp pilins, we examined if their products were modified by the N. gonorrhoeae *O*-glycosylation system. Using immunoblotting with the monoclonal antibody (MAb) npg1, which recognizes a glycan-associated epitope, we found that the PilEs from the N. meningitidis, N. lactamica, and N. polysaccharea 1 isolates were glycosylated, while those from the remaining species showed either no reactivity or weak reactivity in the cases of PilEs from N. polysaccharea 2, *N. mucosa*, and *N. elongata* (Fig. 2, bottom panel). To estimate the relative glycosylation levels for the latter proteins, we performed immunoblotting using serial dilutions of whole cells from a *pglA* null background as standards. By comparing their immunoblot signal intensity to that of the N. gonorrhoeae PilE standard at various dilutions, glycosylation levels were estimated to be approximately 5% for *N. mucosa* and 10 to 15% for *N. elongata* (see Fig. S2 in the supplemental material).

10.1128/mbio.03797-21.2 FIG S2 Estimation of glycosylation levels for *N. elongata* and *N. mucosa* PilEs when expressed in N. gonorrhoeae. Equal amounts of whole-cell lysates from strains expressing PilE from N. gonorrhoeae (strain CH114), *N. elongata* (strain CH4), and *N. mucosa* (strain CH36) were probed against a glycan-specific MAb. For standardization, whole-cell lysate from the strain expressing gonococcal PilE was serially diluted (2-fold). N. gonorrhoeae PilE is 100% glycosylated (i.e., one glycan per PilE monomer). Based on signal intensities, glycosylation levels of PilE pilins from *N. elongata* and *N. mucosa* correspond to approximately 10 to 15% and 5% glycosylation, respectively. Download FIG S2, TIF file, 3.5 MB. Copyright © 2022 Hadjineophytou et al. 2022 Hadjineophytou et al. https://creativecommons.org/licenses/by/4.0/ This content is distributed under the terms of the Creative Commons Attribution 4.0 International license.

Mass spectrometry (MS) analyses of PilE from enriched shear extracts identified glycopeptides for all detectably glycosylated pilins, save for those from *N. mucosa* and N. polysaccharea 2 (see Fig. S3 in the supplemental material). The glycopeptides found for N. meningitidis, N. lactamica, and N. polysaccharea PilEs included conserved serine attachment sites defined earlier for N. meningitidis PilE, for which there was clear evidence for multiple modified residues (23). Despite its low level of glycosylation, a glycopeptide for *N. elongata* PilE was identified, and the residues in this peptide resemble LCR attachment sites identified in N. gonorrhoeae and other broad-spectrum systems (Fig. 1; Fig. S3). PilE glycosylation was also assessed by immunogold labeling/transmission electron microscopy of Tfp, where the results were concordant with those from immunoblotting and MS studies (Fig. S1C).

To examine the degree of glycosylation macroheterogeneity occurring in gonococci, the relative mobilities of pilins in backgrounds expressing tri-, di-, and monosaccharide glycoforms along with a *pgl* null control were assessed (see Fig. S4 in the supplemental material). As seen by the stepwise, retarded migration associated with glycan presence and increasing glycan mass, PilEs from N. meningitidis, N. lactamica, and N. polysaccharea 1 were completely glycosylated. In contrast, migration variation was absent for the other PilEs, save for that from *N. elongata*, where a low level with altered migration was found that corresponded with its reduced glycosylation status detected by glycan MAb reactivity.

10.1128/mbio.03797-21.4 FIG S4 PilE protein glycosylation status and macroheterogeneity assessed by relative mobility shifts in SDS-PAGE. Exogenous *pilE* alleles were expressed in various N. gonorrhoeae glycoform-expressing backgrounds, and corresponding whole-cell extracts were probed with an antiserum raised against gonococcal PilE isolated from a disaccharide-expressing background. Each lane is labeled by the background, which corresponds to different glycoforms expressed in each strain: tri, trisaccharide/diNAcBac-Gal-Gal; di, disaccharide/diNAcBac-Gal; mono, monosaccharide/diNAcBac; −, glycosylation null. The strains used are (from left to right) CH2 to -37, CH76 to -79, KS101, and KS105. Download FIG S4, TIF file, 2.7 MB. Copyright © 2022 Hadjineophytou et al. 2022 Hadjineophytou et al. https://creativecommons.org/licenses/by/4.0/ This content is distributed under the terms of the Creative Commons Attribution 4.0 International license.

### Phylogeny and diversity of *pglO* across the genus *Neisseria*.

**FIG 3:**
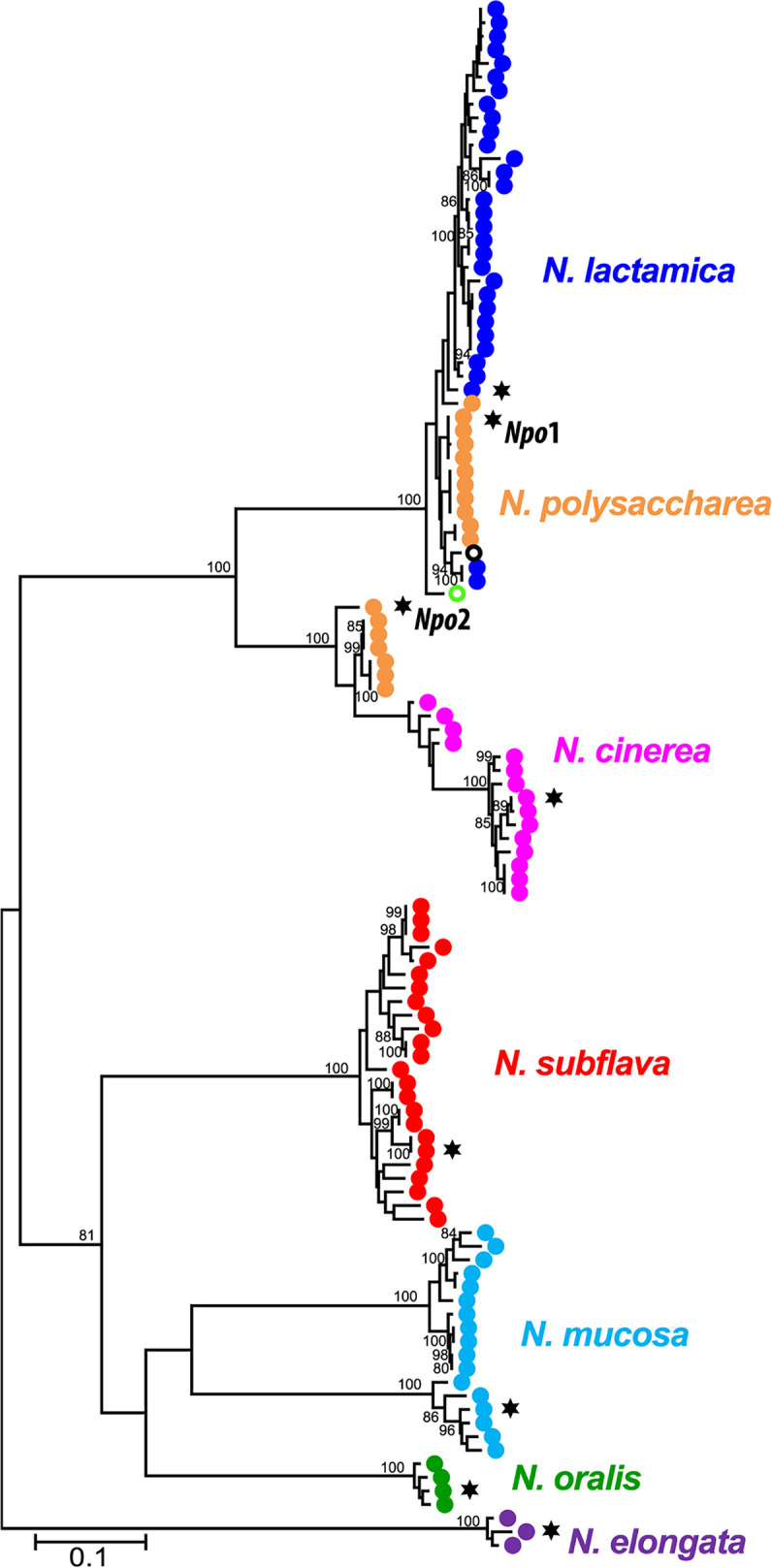
Maximum likelihood phylogenetic tree of neisserial *pglO* alleles. The tree was based on a MUSCLE alignment (66) and was constructed in MEGA X (67) using the Tamura-Nei model (68). Species-representative alleles from N. gonorrhoeae MS11 and N. meningitidis FAM18 were included for reference (green and black open circles, respectively). Alleles of *pglO* expressed in N. gonorrhoeae for the glycosylation complementation experiments are marked with a star. The tree was inferred using 114 sequences at 2,052 sites and replicated 500 times. Bootstrap values with <80% confidence are excluded from the final figure. The strains used can be found in Table S1C.

The ability of *N. elongata* PilE to be glycosylated (albeit inefficiently) in N. gonorrhoeae but not in *N. elongata* (18) suggested that factors independent of PilE structure might impact glycosylation propensity and that this situation might relate to varying *O*-OTase targeting activity. To test this directly, we first identified candidate *O*-OTase genes in neisserial genomes in PubMLST by BLASTp and BLASTn analyses using N. gonorrhoeae PglO/*pglO* and N. meningitidis PglL/*pglL* as queries. (Note that despite the distinct nomenclature, *pglO* and *pglL* are orthologous [4, 24], so we use the terminology PglO/*pglO* here.) Single, quality hits were identified in the genomes of all human-associated neisserial isolates, and analyses of these revealed patterns of phylogenetic relationships congruent with those established using other common gene sets, including protein glycosylation (*pgl*) genes (25–27) (Fig. 3). Interestingly, closer examination revealed two allelic isoforms in N. gonorrhoeae defined by the variable presence of a single nucleotide polymorphism (SNP) in the genus-wide consensus stop codon that results in an ORF that is C-terminally extended by 38 amino acids (see Fig. S5A in the supplemental material). This SNP variant appears to have been acquired multiple times across lineages and is present in the gonococcal background used in studies here (see Fig. S6 in the supplemental material). We corrected the SNP to regenerate the stop codon in the strain used here but found no gross differences in either PilE glycosylation or glycoprotein profile determined by immunoblotting using a glycan-specific MAb (Fig. S5B).

10.1128/mbio.03797-21.5 FIG S5 Inferred consequences of a stop codon SNP on gonococcal PglO structure. (A) The diagram illustrates the extended *pglO* ORF imparted by the TGA-CGA SNP (marked by a star) that results in disruption of the consensus translational stop codon. (B) Glycan-detecting immunoblot of whole-cell lysates of a mutant in which the consensus stop codon has been restored in a strain originally bearing the Arg605 CGA SNP (strain CH101). WT denotes the extract derived from the parental strain used for the construction of the mutant (strain KS127). Download FIG S5, TIF file, 0.7 MB. Copyright © 2022 Hadjineophytou et al. 2022 Hadjineophytou et al. https://creativecommons.org/licenses/by/4.0/ This content is distributed under the terms of the Creative Commons Attribution 4.0 International license.

10.1128/mbio.03797-21.6 FIG S6 Distribution of a stop codon loss SNP in gonococcal *pglO* genes. A midpoint-rooted recombination-corrected maximum likelihood phylogeny of 4,852 genomes based on 68697 nonrecombinant SNPs nonrecombinant from reference 3 was annotated with the presence of the *pglO* ORF-extending SNP (in turquoise, while dark green denotes the consensus wild-type stop codon) identified using variant calls from Pilon v1.16 after mapping WGS reads to NCCP11945 (NC_011035.1) with BWA-MEM v0.7.17-r1188. Branch length represents total number of substitutions after removal of the predicted recombination. Download FIG S6, TIF file, 0.8 MB. Copyright © 2022 Hadjineophytou et al. 2022 Hadjineophytou et al. https://creativecommons.org/licenses/by/4.0/ This content is distributed under the terms of the Creative Commons Attribution 4.0 International license.

### Oligosaccharyltransferases from *Neisseria* species support broad-spectrum glycosylation but vary in PilE targeting proficiency.

**FIG 4:**
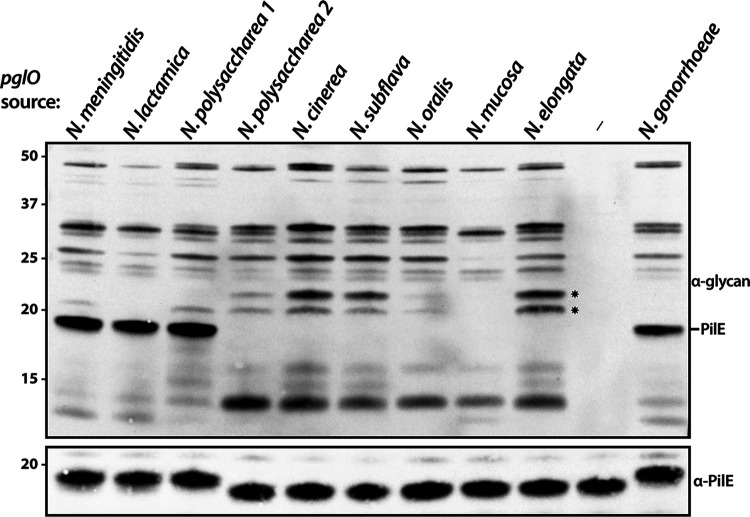
Oligosaccharyltransferases from *Neisseria* species support broad-spectrum glycosylation when expressed in N. gonorrhoeae. (Top panel) Samples probed with a glycan-specific monoclonal antibody. (Bottom panel) The same samples as in the top panel probed with a polyclonal antiserum raised against purified N. gonorrhoeae PilE. The strains used are (from left to right) CH66, CH65, CH63, CH64, CH59, CH60, CH62, CH61, CH48, CH47, and KS127. Two asterisks denote glycoproteins chosen for further analyses (Fig. 5).

To examine the functionality of PglO from diverse species groups, alleles from the same backgrounds from which *pilE* alleles were derived were introduced into the N. gonorrhoeae strain using an allelic replacement protocol. As indicated by the large number of reactive glycoproteins detected via immunoblotting using a glycan-specific MAb, all *pglO* alleles supported broad-spectrum protein glycosylation (Fig. 4). In addition to the expected targeting of endogenous PilE by PglO from both N. gonorrhoeae and N. meningitidis, pilin glycosylation was also seen for the N. lactamica and N. polysaccharea 1 PglOs, but not from the remaining species (Fig. 4).

### Identification of additional glycoproteins differentially modified by N. gonorrhoeae and *N. elongata O*-OTases.

**FIG 5:**
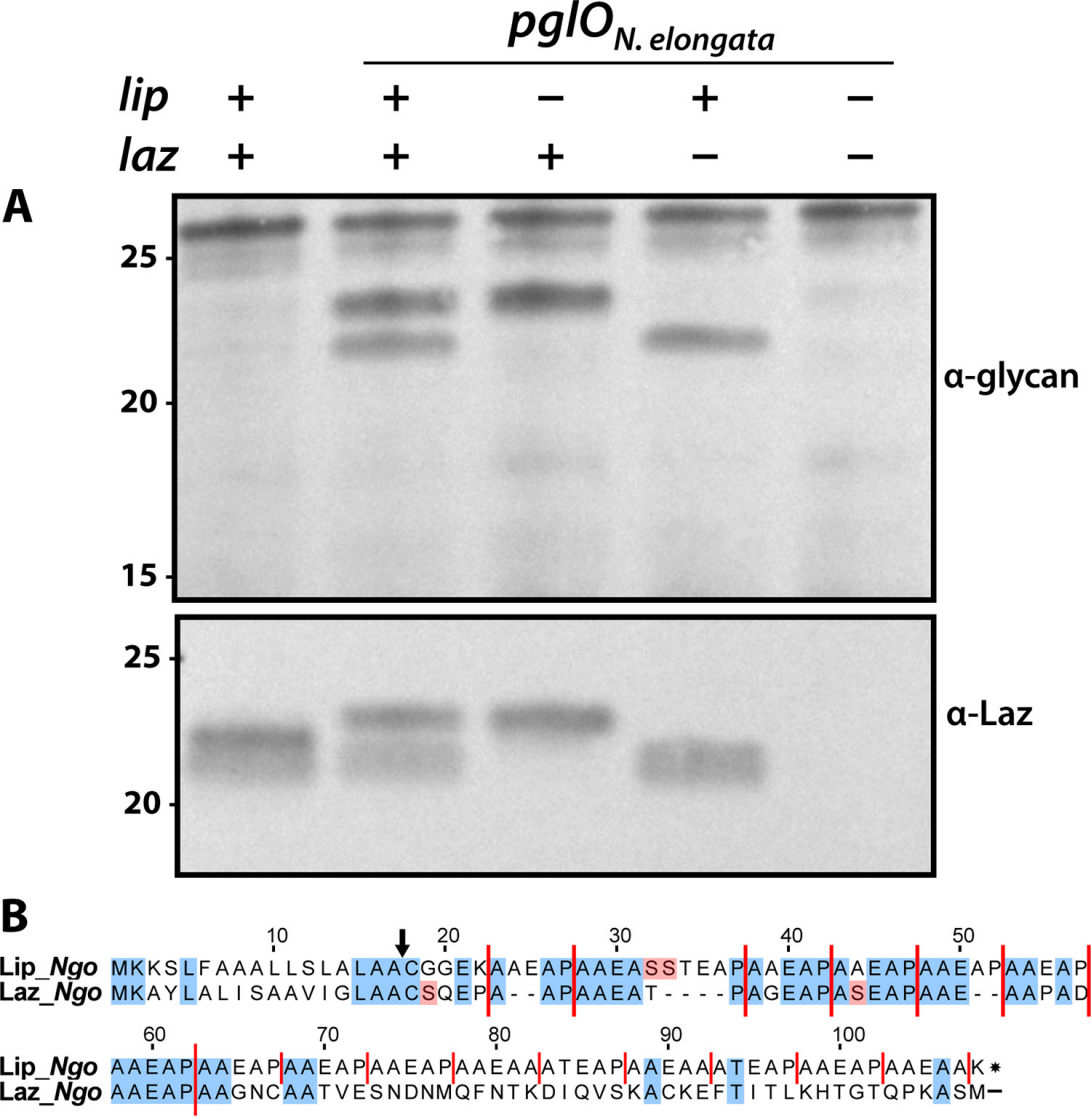
The structurally related lipoproteins Lip and Laz are differentially modified by *N. elongata* versus N. gonorrhoeae *O*-OTases. Shown are immunoblots of N. gonorrhoeae strains expressing either the endogenous *pglO* allele or the *N. elongata pglO* allele along with ORF-inactivating mutations in *lip* and/or *laz*. Samples were probed with either a glycan-recognizing MAb (top panel) or a polyclonal antiserum raised against recombinant Laz purified from E. coli (bottom panel). The latter antibodies react with both Lip and Laz. The strains used are (from left to right) KS127, CH48, CH156, CH155, and CH157. (B) Alignment of Lip and the amino terminus of Laz from N. gonorrhoeae. Conserved residues are highlighted in blue, and serine residues (potential sites of glycan attachment) are highlighted in red. Vertical red lines define AEAAP pentapeptide repeat units (and degenerate forms thereof). The vertical black arrow shows the site of N-terminal proteolytic cleavage of the diacylglyceryl-prolipoprotein.

We next sought to identify other resident proteins differentially glycosylated by *O*-OTases when expressed in *N. gonorrheae*. We focused on two candidates with relative mobilities of 22 kDa and 23 kDa that were strongly reactive with the glycan MAb in the *N. elongata* and other *O*-OTase backgrounds (marked with asterisks in Fig. 4). Based on previous knowledge of N. gonorrhoeae glycoproteins, we identified these as the lipoproteins Ngo0983 (known as Lip) and Ngo0994 (known as Laz [for lipid-linked azurin]) by virtue of their immunoblot reactivity with antibodies raised against recombinant Laz protein (Fig. 5A). Earlier studies showed that although each of these was a glycoprotein, Lip and Laz are poorly glycosylated in N. gonorrhoeae (as shown by their failure to show glycosylation-dependent shifts in relative mobilities) (17, 28). Notably, Lip and Laz lipoproteins share limited sequence identities, other than highly conserved amino-terminal domains rich in pentapeptide (AAEAP) repeats that also encompass serine residues implicated as sites of glycan occupancy (Fig. 5B).

**FIG 6:**
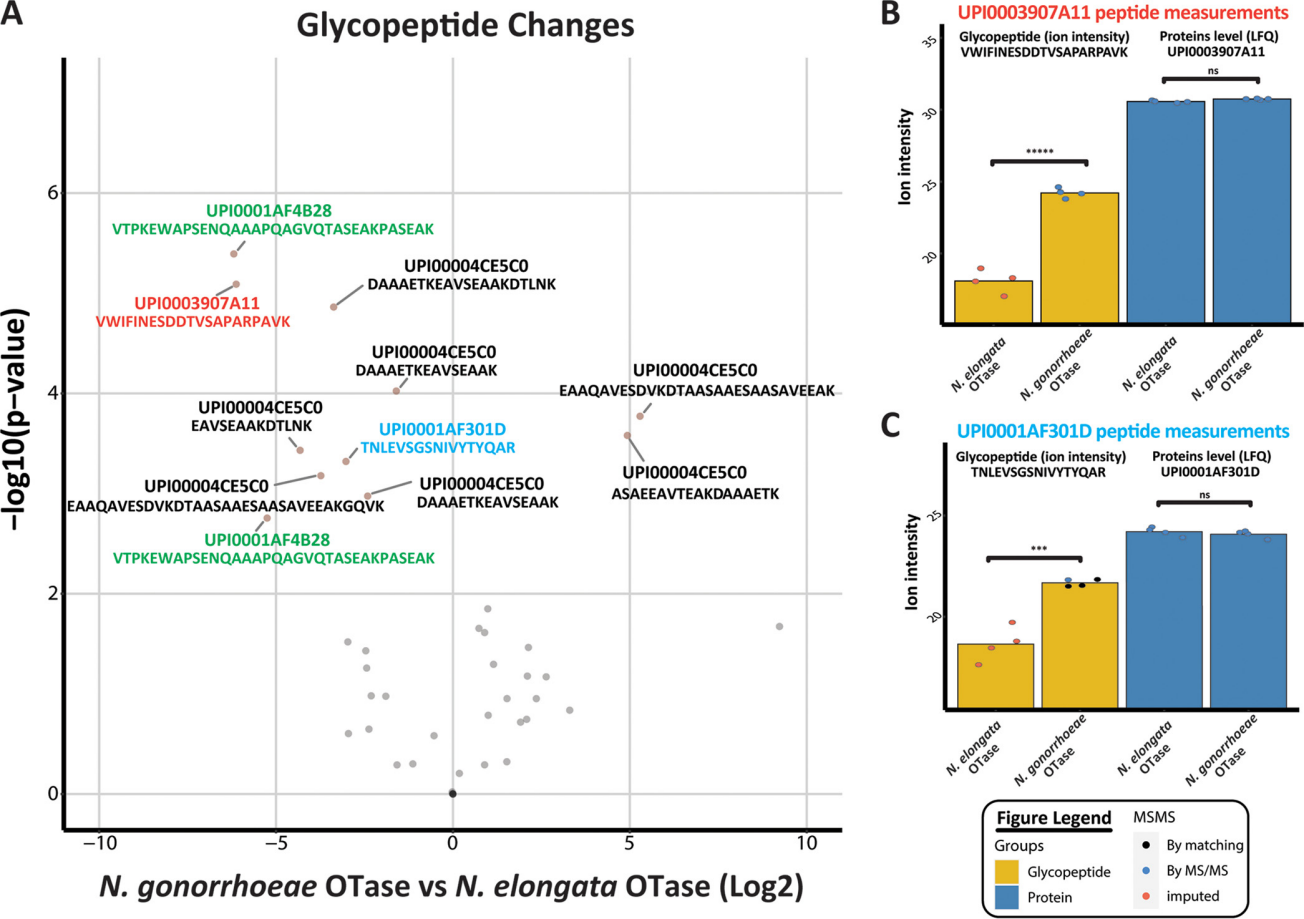
Glycoproteomic/proteomic analysis supports the alteration of glycosylated substrates independent of changes in protein abundance. (A) A volcano plot of quantified glycopeptides reveals alterations in the abundances of glycopeptides within samples expressing different OTases. Examination of peptides identified for the proteins PilQ (UPI0003907A11 [red]) and PilV (UPI0001AF301D [blue]) reveals glycopeptides of these proteins are only observed within strains expressing the N. gonorrhoeae OTase. Other glycopeptides showing significant differences in the two backgrounds are derived from MtrC (UPI0001AF4B28 [green]) and Ag473 (UPI00004CE5C0 [black]). Further analyses of the nonglycosylated peptides derived from PilQ and PilV reveal similar levels of protein abundances in both backgrounds (panels B and C, respectively).

We also employed an unbiased, MS-based methodology based on high-field asymmetric waveform ion mobility spectrometry (FAIMS) fractionation (29) to compare the whole proteomes and glycoproteomes of N. gonorrhoeae expressing either the endogenous *O*-OTase or that from *N. elongata*. Here, alterations in the abundances of numerous glycopeptides were noted, including those derived from the glycoproteins Ag473 (Ngo1043), the secretin PilQ, the multidrug efflux transporter component MtrC, and the minor pilin subunit PilV (Fig. 6A). Changes in glycopeptide detection here could reflect alterations in the abundance of the proteins themselves from which they are derived. In the cases of PilQ and PilV, however, proteomic analysis showed that these differences could not be accounted for by such alterations in overall protein abundance (Fig. 6B and C). Further analyses nonetheless revealed that there were clear differences in the relative abundances of a large number of other proteins between samples derived from the two PglO backgrounds (see Fig. S7 in the supplemental material).

10.1128/mbio.03797-21.7 FIG S7 Expression of the *N. elongata* PglO allele leads to changes within the N. gonorrhoeae proteome. (A) Pearson correlation analysis of proteomic samples reveals high similarity within the proteomes of N. gonorrhoeae N400 expressing either the N. gonorrhoeae or *N. elongata O*-OTase, which highlights replicates expressing each group tightly together. (B) Principal-component analysis (PCA) supports the tight clustering of biological replicates within replicates from the different OTases. (C) Volcano plot of the proteomic changes multiple proteins undergo with profound alterations in response to expression of different PglO proteins. (D) Heat map of proteins observed with an absolute fold change of >2 log_2_ and *P* value of <0.01 reveals a high degree of consistency within replicates expressing the *N. elongata* PglO compared to N. gonorrhoeae PglO. Download FIG S7, TIF file, 2.3 MB. Copyright © 2022 Hadjineophytou et al. 2022 Hadjineophytou et al. https://creativecommons.org/licenses/by/4.0/ This content is distributed under the terms of the Creative Commons Attribution 4.0 International license.

### Chimeric *O*-OTases reveal domains influencing PilE targeting specificity.

**FIG 7:**
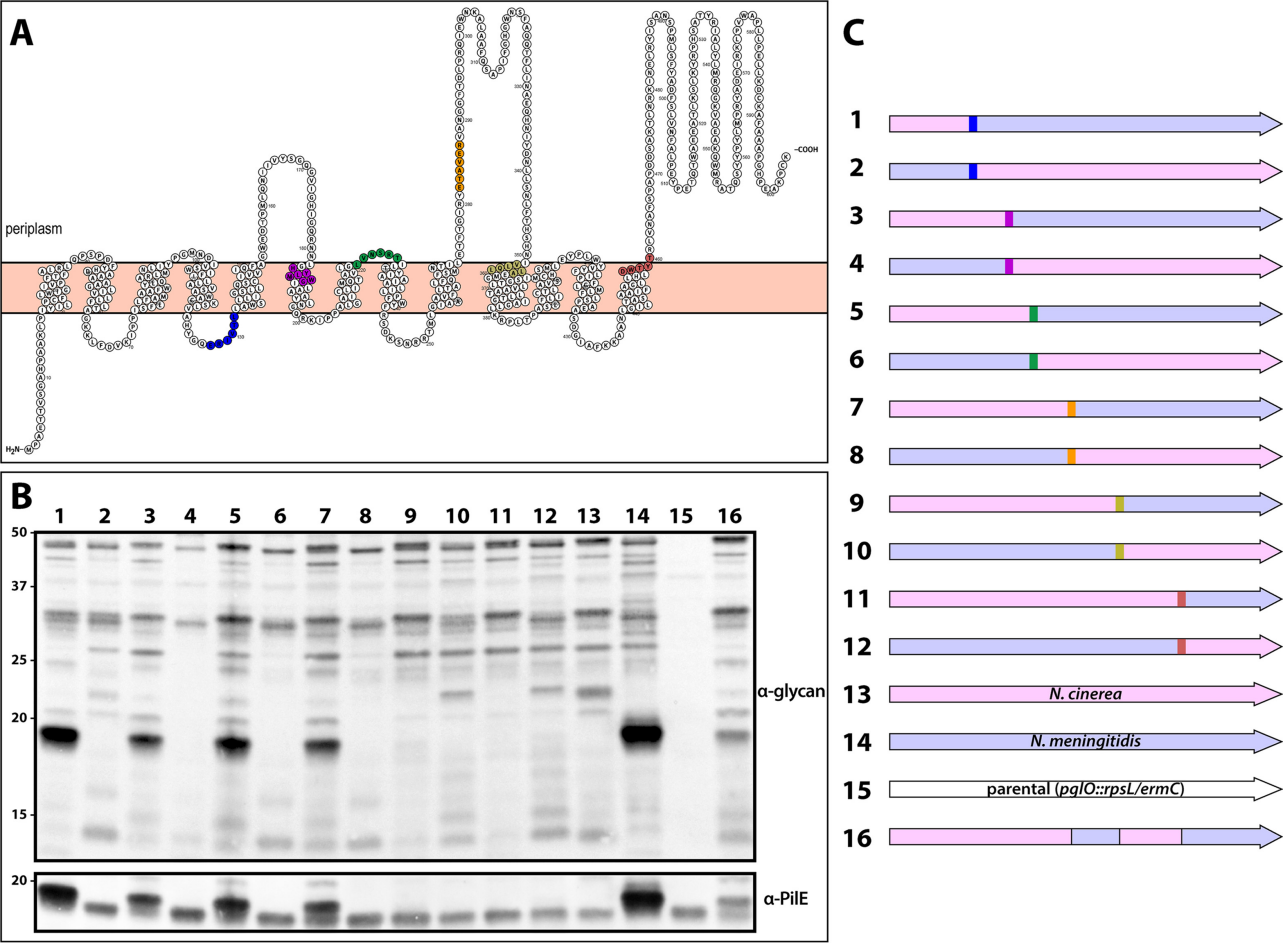
Chimeric *O*-OTases reveal domains influencing PilE targeting specificity. (A) Predicted transmembrane topologies of N. meningitidis PglO based on Phobius (69) and visualized using Protter (70). Color-coded stretches indicate conserved amino acid residues shared between N. meningitidis and *N. cinerea* alleles that were used as fusion sites. (B) Immunoblot of whole-cell lysates of N. gonorrhoeae strains expressing *pglO* chimeric constructs. (Top panel) Membrane probed with a glycan-specific MAb. (Bottom panel) The same membrane shown in top panel reprobed with the PilE-recognizing polyclonal antiserum. (C) Cartoon illustration of the PglO chimeras color coded so as to delineate fusion sites as shown in panel A. The numbering here corresponds to the lane numbers in panel B. The strains used are (from left to right in panel B) CH91 to -100, CH105, CH106, CH59, CH66, CH47, and CH152.

To begin to assess structural features of *O*-OTases associated with distinct substrate protein targets, we sought features that might explain their abilities to differentially glycosylate PilE. Consensus models from membrane protein structure algorithms predicted that all neisserial PglOs have similar topologies, with 13 transmembrane domains and three large extrahelical loops oriented toward the periplasm (see Fig. S8A in the supplemental material). These patterns mirrored those of the *N-*linked PglB OTase from Campylobacter lari (11) and other members of SEDS family proteins (2). Comparative sequence alignment of neisserial PglOs revealed multiple regions of interspecies diversity, but no obvious patterns were correlated with their observed activities on PilE (Fig. S8B, top panel). Therefore, we set out to identify domains responsible for PilE targeting by creating hybrids between the two most closely related *O*-OTases that differed in PilE targeting activities. To this end, we created chimeras by fusing ORF-encoding sequences from the *O*-OTases of N. meningitidis and Neisseria cinerea that share 69.9% identity and 75.1% similarity (Fig. S8B, bottom panel). Sites of fusion were targeted to highly conserved stretches of amino acids (Fig. 7: Fig. S8B, top panel). The chimeras were then expressed in N. gonorrhoeae via allelic replacement and their targeting activities assessed by immunoblotting using glycan-recognizing antibodies.

10.1128/mbio.03797-21.8 FIG S8 (A) Conserved topological features of neisserial oligosaccharyltransferases. Membrane topologies were inferred using the TOPCONS hidden Markov model, which predicts the final protein transmembrane profile based on the consensus generated from five different topology prediction algorithms (4). The predicted topologies are color coded as follows: red, cytoplasmic; blue, periplasmic; gray box, transmembrane domain leading to the periplasm; white box, transmembrane domain leading to the cytoplasm. (B) Sequence relationships of PglO proteins used in comparative studies. (Top panel) Residues shared by ≥80% of the proteins are highlighted in purple. Pfam domains (as predicted by HMMER) are underlined as follows: red, PF15864 (PglL_A); black, PF04932 (Wzy_C); and green, PF11846 (Wzy_C_2). Sequences were aligned using MUSCLE and visualized with Jalview v2.11. (Bottom panel) Identity and similarity-based matrices of PglO proteins examined. The panel was generated using the alignment shown in top panel and visualized in Excel. Download FIG S8, TIF file, 1.8 MB. Copyright © 2022 Hadjineophytou et al. 2022 Hadjineophytou et al. https://creativecommons.org/licenses/by/4.0/ This content is distributed under the terms of the Creative Commons Attribution 4.0 International license.

All chimeras glycosylated multiple gonococcal proteins, although each was associated with distinct patterns of protein targeting (Fig. 7B and C). PilE glycosylation was only observed for hybrids carrying C-terminal segments of N. meningitidis PglO. This activity was retained in hybrid 7, which carried the last 317 residues of N. meningitidis PglO, but it was absent from hybrids 9 and 11, which carried the last 247 and 144 residues, respectively. Based on these results, we hypothesized that the PilE targeting specificity might minimally require the two major, putative periplasmic domains of N. meningitidis PglO. Indeed, a hybrid consisting of an *N. cinerea* PglO backbone carrying solely these two segments from N. meningitidis PglO was capable of glycosylating PilE (Fig. 7).

## DISCUSSION

Broad-spectrum protein glycosylation systems are found in all branches of life. Despite progress in a number of systems, there remain significant gaps in knowledge as to what factors shape glycoproteome content and how protein-targeting transferases select their substrates. Addressing these shortcomings seems crucial to understanding the biological significance of protein glycosylation at both the molecular and evolutionary levels. As shown in this study, the *O*-linked glycosylation systems expressed by human-associated species groups of the genus *Neisseria* have high potential to address both of these questions. We are unaware of other studies examining OTase structure-function diversity at this level. In fact, with the notable exception of dedicated *O*-OTases, the general consensus from limited studies of *O*-OTases in *Burkholderia* and Acinetobacter isolates and *N*-OTases in Campylobacter species and related Deltaproteobacteria/*Epsilonproteobacteria* members seems to be that highly structurally related OTases have similar targeting activities, respectively (30–33).

The restricted distribution of the PilE glycosylation phenotype (and underlying genotypes) within neisserial species groups raises questions as to its biological, evolutionary, and ecological significance. In gonococci, PilE glycosylation can influence cell-cell interactions occurring within microcolonies that likely stem from subtle changes in pilus extrusion-retraction dynamics and biophysical properties of pili themselves (34). Glycan modification has also been suggested to act as a cloaking device to mask conserved PilE protein epitopes or redirect the humoral response toward the glycan, which in and of itself is antigenically variable. Such scenarios have been particularly invoked in the case of the structurally invariant class II pilins expressed by some meningococcal isolates (23). In addition, disrupted glycosylation of some PilE variants in N. meningitidis was associated with a dramatic decrease in the levels of Tfp (23). Pilin subunit glycosylation in P. aeruginosa has also been implicated in having an impact on Tfp expression levels and organelle dynamics, as well as blocking predation by bacteriophages that use Tfp as primary receptors (35, 36). Filamentous phages using Tfp as primary receptors have been described in gonococci and meningococci, although there are no reports that PilE glycosylation influences phage susceptibility (37, 38). Thus, while the basis for the variable distribution of PilE glycosylation remains to be determined, we speculate that it involves differences in the roles Tfp serve in niches occupied by distinct neisserial species groups.

A point of interest with regard to PilE glycosylation is its association with particular glycoforms and *pgl* glycosyltransferase genes. Specifically, distribution of PilE glycosylation coincides remarkably with that of the *pglA* and *pglE* glycosyltransferase genes that incorporate specifically galactose sugars into di- and trisaccharide glycoforms (26). Terminal galactose on PilE-associated glycans has been proposed to promote adherence to human cervical mucosa via an interaction with the I-domain of complement receptor 3 (39). Another connection between PilE glycosylation and glycan expression entails the correlation with phase-variable glycosyltransferase alleles whose on-off expression underlies glycan antigenic variation (40, 41, 71). Such phase-variable alleles of *pglA*, *pglG*, and *pglH* are uniquely restricted to species isolates defined here as capable of the PilE glycosylation phenotype (27). As abundant surface-exposed glycoproteins and critical colonization factors in N. gonorrhoeae and N. meningitidis (and likely in N. polysaccharea and N. lactamica), PilE glycosylation and glycan antigenic variation may have coevolved as synergistic mechanisms to evade immune surveillance of Tfp, as suggested by others (23).

The distinct substrate targeting specificities and relatively high degrees of sequence identity exhibited by neisserial PglOs appear to provide a unique set of reagents to define structure-function relationships of *O-*OTases. While the potential of the neisserial system in this regard is exemplified by the findings using PglO chimeras, it is also of interest to define the structural features of protein targets that influence glycosylation susceptibility and productive interaction. That prospect is supported here by the finding that Lip and Laz as two proteins coordinately and differentially glycosylated by PglO from *N. elongata* share only a short segment of amino acid identity restricted to their N termini (Fig. 5). It should be possible then to apply the polypeptide chimera approach used in the PglO analyses to assess and define key features of protein substrates as well. Further analyses of the glycopeptide profiles and glycoproteomes associated with distinct PglO isoforms should also provide valuable information on the details of substrate-OTase interactions. That said, we cannot formally rule out that the PglO targeting specificities seen here may be influenced by the efficacies with which exogenous *O*-OTases use gonococcal glycan donors.

In conclusion, neisserial *O*-OTases and their distinct targeting activities provide a unique and facile set of naturally occurring reagents to address fundamental questions about the molecular mechanisms by which proteins are targeted for glycosylation as well as the evolution of protein glycosylation systems.

## MATERIALS AND METHODS

### Bacterial strains and culture conditions.

*Neisseria* strains were grown for 16 to 18 h (at 37°C with 5% CO_2_) on GC medium (Difco), supplemented with Kellogg’s supplement (42). The gonococcal strains used were derivatives of strain MS11 carrying an isopropyl-β-d-thiogalactopyranoside (IPTG)-inducible *pilE* allele (43). Gonococcal transformants were selected on antibiotics at the following concentrations: streptomycin, 750 μg/mL; kanamycin, 50 μg/mL; and chloramphenicol, 10 μg/mL. Genetic constructs and mutations in glycosylation genes (*pglE*_on_, *pglA*, and *pglC*) were introduced into various strains using transformation as previously described (44). Escherichia coli TOP10 cells (Invitrogen) were used for plasmid propagation. The strains, plasmids, and primers used in this study are listed in Table S1A and B in the supplemental material.

### Genome analyses and bioinformatics.

Candidate *pilE* and *pglO* genes were identified using BLASTp and BLASTn queries with default parameters against all human-specific *Neisseria* spp. deposited in the Bacterial Isolate Genome Sequence Database (BIGSdb) (http://pubmlst.org/neisseria/) (45). The list of strains utilized is in Table S1C.

### Expression of exogenous *pilE* alleles in *Neisseria*.

The *pilE* alleles from selected *Neisseria* species were ectopically expressed in an intergenic region of N. gonorrhoeae as translational fusions to the native N. gonorrhoeae *pilE* at their conserved Gly_−1_Phe_+1_ junction (see Fig. S9 in the supplemental material) as previously described (46, 47). This was achieved by PCR amplifying the two overlapping fragments, followed by PCR-based splicing by overhang extension (SOE) using the flanking primers. The hybrid constructs were digested with restriction enzymes PacI and FseI and ligated within the intergenic chromosomal site between the *lctP* and *aspC* genes of N. gonorrhoeae in plasmid pGCC6 (48). The plasmids were then linearized and transformed into gonococcal backgrounds in which the endogenous *pilE* allele is placed under an IPTG-inducible promoter (KS101-derived strains). N. gonorrhoeae strains expressing exogenous *pilE* alleles were selected on chloramphenicol-containing media.

10.1128/mbio.03797-21.9 FIG S9 Genetic constructs and mutagenesis strategies. (A) Putative *pilE* alleles from human-restricted *Neisseria* species were expressed as translational fusions with N. gonorrhoeae MS11 *pilE* at the conserved Gly_−1_Phe_+1_ residue. (B) The N. gonorrhoeae *pglO* allele was replaced with a selectable (*ermC*) and counterselectable (*rpsL*) marker, which was then exchanged with *pglO* alleles from selected *Neisseria* strains. The extended *pglO* ORF from N. gonorrhoeae N400 was shortened by 83 bases by introducing the consensus SNP that changes the Arg605 residue to a stop codon using the same allelic exchange strategy. Download FIG S9, TIF file, 0.3 MB. Copyright © 2022 Hadjineophytou et al. 2022 Hadjineophytou et al. https://creativecommons.org/licenses/by/4.0/ This content is distributed under the terms of the Creative Commons Attribution 4.0 International license.

### Quantitative transformation assays.

Transformation assays were performed as previously described (43). Briefly, *gyrB*-containing DNA was PCR amplified from plasmid pSY6 using primers CHP80 and CHP81 (47). Then, 0.1 μg of purified DNA was mixed with a bacterial suspension in 0.5 mL of transformation medium (CO_2_-saturated GC medium supplemented with IsoVitaleX and 7 mM MgCl_2_) and incubated at 37°C for 20 min in the presence of 5% CO_2_. This was followed by a 5-fold dilution with transformation medium and transfer in a 37°C shaking incubator for 3 h. Finally, cells were appropriately diluted and plated onto GC plates with and without nalidixic acid. Transformation frequencies were calculated by dividing the number of nalidixic acid-resistant CFU by the total CFU.

### Allelic exchange of the *pglO* locus in N. gonorrhoeae.

Various neisserial *pglO* alleles were introduced into N. gonorrhoeae via a two-step mutagenesis strategy that allows markerless gene replacement (Fig. S9) by exploiting the fact that the N. gonorrhoeae MS11 strain is naturally resistant to streptomycin. This method utilizes a two-gene cassette that contains a selectable marker (*ermC*) and counterselectable marker (*rpsL*) (49). First, the immediate flanking regions of *pglO* from N. gonorrhoeae strain were PCR amplified using primers CHP105 and CHP60 for the upstream fragment and CHP191 and CHP56 for the downstream fragment. Then, the *rpsL*_*ermC* locus was PCR amplified from plasmid pFLOB4300 (49) with primers CHP66 and CHP67, which include sequence homology to the flanking regions of the *pglO* allele. The three fragments were PCR purified and spliced in a PCR using the flanking primers CHP105 and CHP56. The product of this reaction was transformed into strain KS127 and selected on erythromycin to create the intermediate strain CH47. Strain CH47 was then used in a second transformation to replace the *rpsL*_*ermC* locus with *pglO* allele ORFs (precisely from the start codon to the stop codon) from different *Neisseria* species, creating strains CH59 to CH67. This was achieved by PCR amplifying the *pglO* ORFs from various species (primers listed in Table S1B) and splicing them in a PCR with the N. gonorrhoeae *pglO* flanking products described above. These *pglO* constructs were cloned into the pCRII-TOPO vector to create pCH59-67, sequenced, and transformed into the intermediate strain CH47. Transformants were selected on streptomycin and screened for erythromycin sensitivity.

For chimeric *O*-OTase constructs, we first identified short stretches of conserved amino acid residues that are shared by both N. meningitidis and *N. cinerea pglO* alleles. These conserved regions were used in primer design to create homologous overlapping fragments between the two alleles. For templates, we used the pCRII-TOPO plasmids pCH66 and pCH59 (described above) to generate two overlapping fragments that were independently PCR amplified, spliced in a new PCR with flanking primers, and cloned in a plasmid. Finally, the constructs were transformed into strain CH47. The primers used were CHP105 and CH56 (described above) in conjunction with the hybrid-specific primers listed in Table S1B.

### Construction strategies for mutants.

The *lip* and *laz* genes from N. gonorrhoeae were inactivated via the insertion of a kanamycin cassette within their ORFs, resulting in the deletion of 83% and 94% of the *lip* and *laz* genes, respectively. First, the kanamycin gene was amplified from pKAN with primers CHP172 and CHP173. To delete *laz*, primer pairs CHP215 and CHP216 and CHP217 and CHP218 were used to amplify the flanking regions of *laz*, which were then spliced in a PCR-based SOE reaction with the kanamycin cassette. The PCR product was TOPO cloned, transformed into CH48, and selected on kanamycin to create strain CH155. Similarly, the flanking regions of *lip* were PCR amplified with primer pairs CHP219 and CHP220 and CHP221 and CHP222 and then spliced with the kanamycin cassette. Finally, the PCR product was TOPO cloned, transformed into CH48 and CH155, and selected on kanamycin to create strains CH156 and CH157.

We used site-directed mutagenesis to introduce a stop codon to the extended *pglO* ORF of N. gonorrhoeae. This was achieved by mutating the Arg605 residue of *pglO* to a stop codon (CGA-TGA). Two PCR fragments were generated using primer pairs CHP105 and CHP213 and CHP212 and CHP56, which carried the SNP in their overlapping homologous regions. The two fragments were spliced in a new PCR using flanking primers CHP105 and CHP56, purified, and cloned into a TOPO vector. Finally, the plasmid was linearized and transformed into CH47, creating strain CH101 (Fig. S9).

### SDS-PAGE, immunoblotting, and pilus purification.

Procedures for SDS-PAGE and immunoblotting have been previously described (17). Briefly, glycoproteins were detected by immunoblotting of whole-cell lysates using rabbit antibodies and alkaline phosphatase-coupled goat anti-rabbit secondary antibodies (Sigma). Rabbit monoclonal antibodies npg1 and npg3 were used at a 1:10,000 dilution to detect di-*N*-acetylbacillosamine (diNAcBac) or diNAcBac-Gal-Gal-carrying glycoproteins, respectively (50). To detect PilE subunits, the rabbit polyclonal antibody 903 was used at a 1:2,500 dilution (51). Rabbit antiserum to the Laz protein was a gift from James Moir (52). Pili were purified by shear extraction in ethanolamine buffer and subsequent ammonium sulfate concentration as previously described (53).

### LC-MS2 analysis of trypsin generated pilin peptides.

Procedures for in-gel digestion was performed as previously described using trypsin (Sigma) (54) with the following adjustments. Destaining of gel pieces was done overnight with Milli-Q water. Generated peptides were Zip-tipped as described by the manufacturer (Merck), and the dried peptides were resuspended in a mixture of 2.5% acetonitrile (Acn) and 0.1% formic acid (FA) prior to liquid chromatography-tandem mass spectrometry (LC-MS2) analysis.

LC-MS2 was performed on a system consisting of a Dionex Ultimate 3000 RSLCnano-LC system (Sunnyvale CA, USA) connected to a hybrid quadrupole Orbitrap (QExactive) mass spectrometer (ThermoElectron, Bremen, Germany) equipped with a nanoelectrospray ion source. For LC separation, an Acclaim PepMap 100 column (C_18_, 3 μm, 100 Å) (Dionex, Sunnyvale CA, USA) capillary of 25-cm bed length was used with a flow rate of 300 nL/min. Two solvents, A (2.5% Acn, 0.1% FA) and B (aqueous 90% Acn in 0.1% FA), were used to elute the peptides from the nano column. Peptide separation were achieved on a gradient from 3% to 55% (solvent B) over 45 min, before a final wash sequence of 90% solvent B for 2 min. The mass spectrometer was operated in the data-dependent mode to automatically switch between MS and MS2 acquisition. Survey full-scan MS spectra (from *m*/*z* 200 to 2,000) were acquired with the resolution *R* = 70,000 at *m*/*z* 200, with an automatic gain control (AGC) target of 1e6. The maximum allowed ion accumulation times were 100 ms. The sequential isolation of up to the seven most intense ions, depending on signal intensity (intensity threshold of 1e4), was considered for fragmentation using higher-energy collisional induced dissociation (HCD) at a target value of 100,000 charges and a resolution *R* = 17,500 with normalized collision energy (NCE) of 28. Target ions already selected for MS/MS were dynamically excluded for 30 s. The isolation window was *m*/*z *= 1.5 without offset. The maximum allowed ion accumulation for the MS/MS spectrum was 180 ms. For accurate mass measurements, the lock mass option was enabled in MS mode for internal recalibration during the analysis.

For the data analysis, the generated MS2 spectra were manually investigated using Qual browser 2.2 (Thermo Scientific) and glycopeptide spectra extracted based on the presence of reporter ions for di-*N*-acetylbacillosamine (diNAcBAc) (at *m*/*z* 229.118 and *m*/*z* 211.1079), diNAcBAc-Gal (at *m*/*z* 391.170), and diNAcBAc-AcGal (at *m*/*z* 433.181). Selected spectra were verified using ProteinProspector (http://prospector.ucsf.edu/prospector/mshome.htm), operated by the UCSF mass spectrometry facility.

### Processing of proteome samples.

Precipitated proteome samples were prepared using S-trap columns (Protifi, USA) according to the manufacturer’s instructions. Briefly samples were resuspended in 5% SDS by boiling, and then protein amounts were quantified using a bicinchoninic acid (BCA) assay (Thermo Fisher Scientific). One hundred micrograms of each sample was then reduced with 10 mM dithiothreitol (DTT) at 95°C for 10 min, allowed to cool to room temperature, and then alkylated with 40 mM chloroacetamide for 30 min in the dark. Samples were then acidified with phosphoric acid to a final concentration of 1.2% and mixed with 7 volumes of 90% methanol–100 mM triethylammonium bicarbonate (TEAB) (pH 7.1) before being applied to S-trap minicolumns. Samples were washed four times with 90% methanol–100 mM TEAB (pH 7.1) to remove SDS, and then 8 μg of trypsin–Lys-c (Promega, USA) in 100 mM TEAB (pH 8.5) was spun through the S-trap columns. Samples were digested for 4 h at 47°C and then collected from the S-traps by washing with 100 mM TEAB (pH 8.5), followed by 0.2% FA, followed by 0.2% FA–50% Acn. Peptide washes were pooled, dried, and then resuspended in buffer A* (0.1% trifluoroacetic acid [TFA], 2% Acn) before being cleaned up with homemade high-capacity StageTips composed of 1 mg Empore C_18_ material (3M) and 5 mg of Oligo R3 reverse-phase resin (Thermo Fisher Scientific, USA) as previously described (55, 56). Columns were wet with buffer B (0.1% FA, 80% Acn) and conditioned with buffer A* prior to use. Resuspended samples were loaded onto conditioned columns and washed with 10 bed volumes of buffer A*, and bound peptides were eluted with buffer B before being dried and then stored at −20°C.

### LC-MS analysis of proteome samples.

StageTip cleaned up samples were resuspended in buffer A* and separated using a two-column chromatography setup composed of a PepMap100 C_18_ 20-mm by 75-μm trap and a PepMap C_18_ 500-mm by 75-μm analytical column (Thermo Fisher Scientific) coupled to a Orbitrap Fusion Lumos Tribrid mass spectrometer equipped with a FAIMS Pro interface (Thermo Fisher Scientific). Gradients of 145 min were run for each sample, altering the buffer composition from 2% buffer B to 28% B over 126 min, then from 28% B to 40% B over 9 min, then from 40% B to 80% B over 3 min, the composition was held at 80% B for 2 min, and then the composition was dropped to 2% B over 2 min and held at 2% B for another 3 min. The Lumos mass spectrometer was operated in a stepped FAIMS data-dependent mode at three different FAIMS compensation voltages (CVs) of −25, −45, and −65 as previously described (57). For each FAIMS CV, a single Orbitrap MS scan (*m*/*z* 350 to 2,000, maximal injection time of 50 ms, and AGC set to a maximum of 4 ×10^5^ ions with a resolution of 60,000) was acquired every 1.35 s, followed by Orbitrap MS/MS HCD scans of precursors (NCE of 30%, maximal injection time of 80 ms, and AGC set to a maximum of 1.25 × 10^5^ ions with a resolution of 30,000). HCD scans containing the oxonium ions (*m*/*z* 204.0867, 138.0545, 366.1396, 229.1189, or 211.1082) triggered three additional product-dependent MS/MS scans (58) of potential glycopeptides: a Orbitrap EThcD scan (NCE of 15%, maximal injection time of 250 ms, AGC set to a maximum of 2 × 10^5^ ions with a resolution of 30,000, and using the extended mass range setting to improve the detection of high-mass glycopeptide fragment ions) (59); a ion trap CID scan (NCE of 35%, maximal injection time of 40 ms, and AGC set to a maximum of 5 × 10^4^ ions) and a stepped collision energy HCD scan (NCE of 35% with 5% stepping, maximal injection time of 250 ms, and AGC set to a maximum of 2 × 10^5^ ions with a resolution of 30,000).

### Proteomic analyses.

Data files were separated into individual FAIMS fractions using the FAIMS MzXML Generator (29) and processed with MaxQuant (v1.6.17.0) (60). Searches were performed against the N. gonorrhoeae MS11 proteome (UniProt accession no. UP000016457 [2,047 proteins]) and the *N. elongata pglO* sequence (UniProt accession no. D4DS59). Searches were undertaken using “Trypsin” enzyme specificity with carbamidomethylation of cysteine as a fixed modification. Oxidation of methionine and the glycan diNAcBAc-Hex2 (chemical composition: C_22_H_36_O_14_N_2_ [552.2166 Da]) were included as variable modifications, and a maximum of 2 missed cleavages was allowed. To enhance the identification of peptides between samples, the “Match between Runs” option was enabled with a precursor match window set to 2 min and an alignment window of 20 min with the label free quantitation (LFQ) option enabled (61). The resulting outputs were processed within the Perseus (v1.6.0.7) analysis environment (62) to remove reverse matches and common protein contaminants prior to further analysis. For LFQ comparisons, biological replicates were grouped, and missing values were then imputed based on the observed total peptide intensities, with a range of 0.3σ and a downshift of 2.5σ using Perseus. Student’s *t* tests were undertaken at the protein and modified peptide levels to compare between groups, and the resulting data were exported to be visualized using ggplot2 (63) within R.

### Data availability.

The MS data and search results have been deposited into the PRIDE ProteomeXchange Consortium repository (64, 65) and can be accessed with the identifier PXD024737.
