# Supplementary figures and images for: Sculpting the Bacterial O-Glycoproteome: Functional Analyses of Orthologous Oligosaccharyltransferases with Diverse Targeting Specificities
Source: mBio. 2022 Apr 26;13(3):e03797-21. doi: 10.1128/mbio.03797-21 (PMC9239064; doi:10.1128/mbio.03797-21)

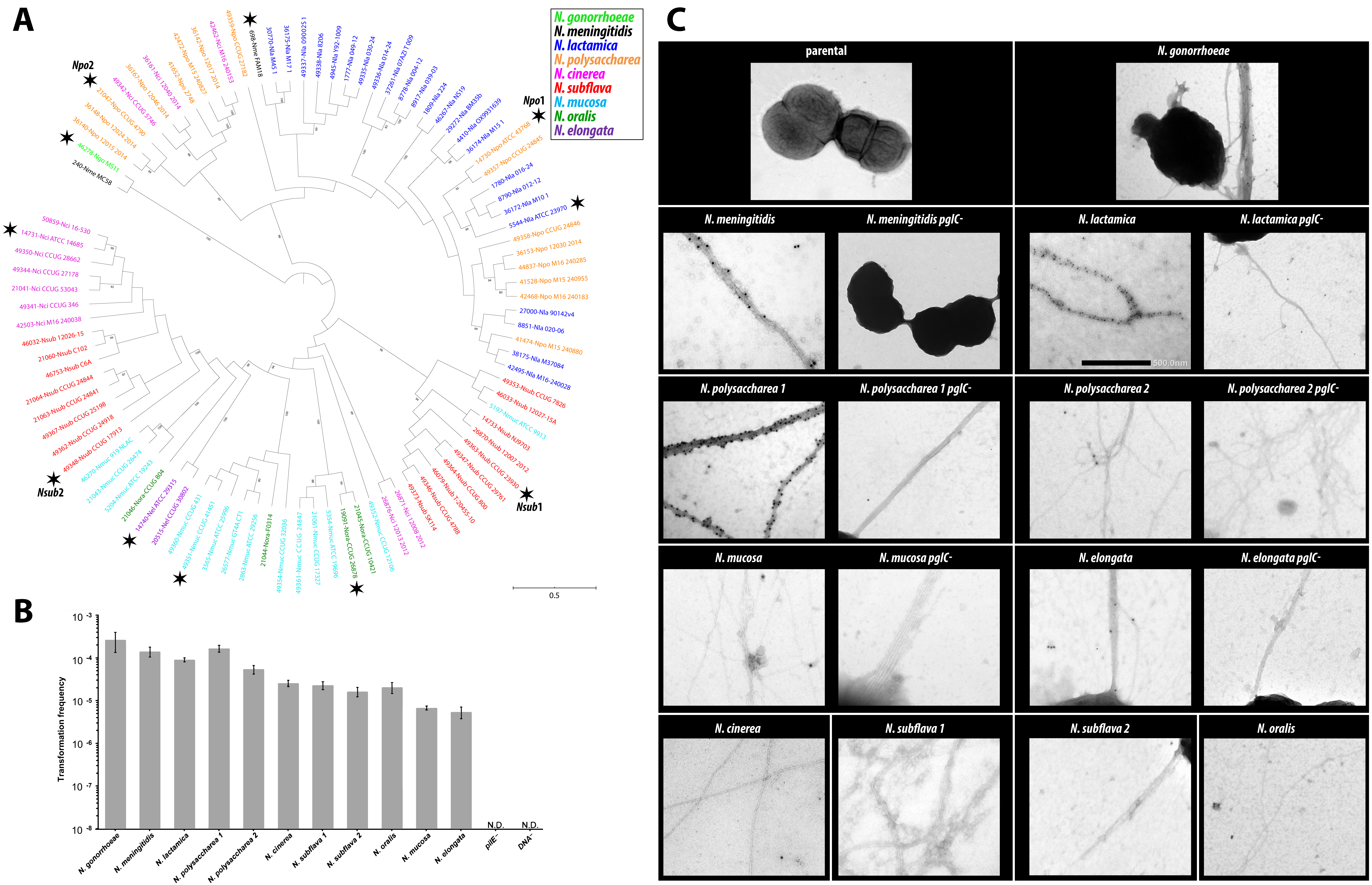

Supplement: FIG S1 [file mbio.03797-21-sf001.tif]

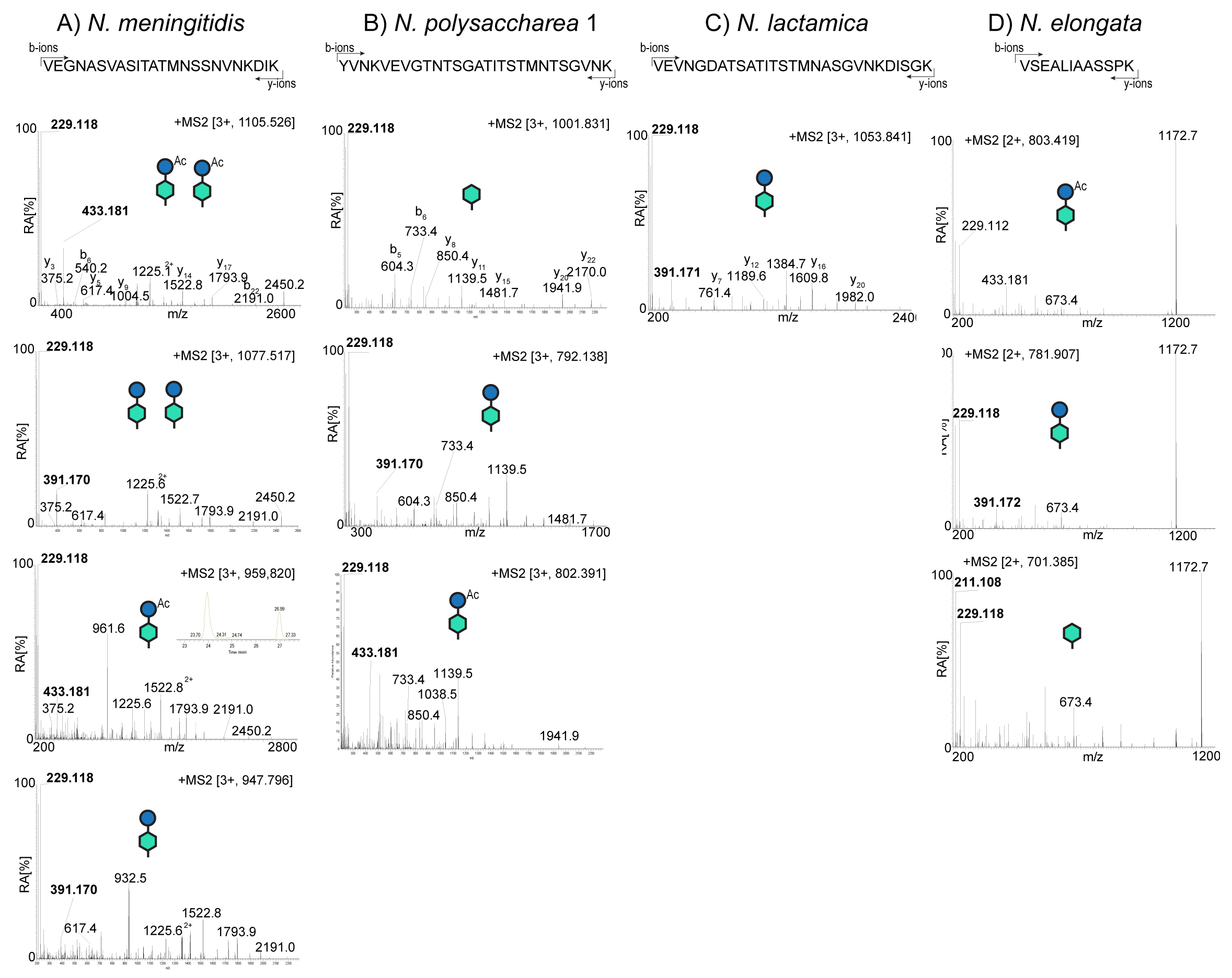

Supplement: FIG S3 [file mbio.03797-21-sf003.tif]

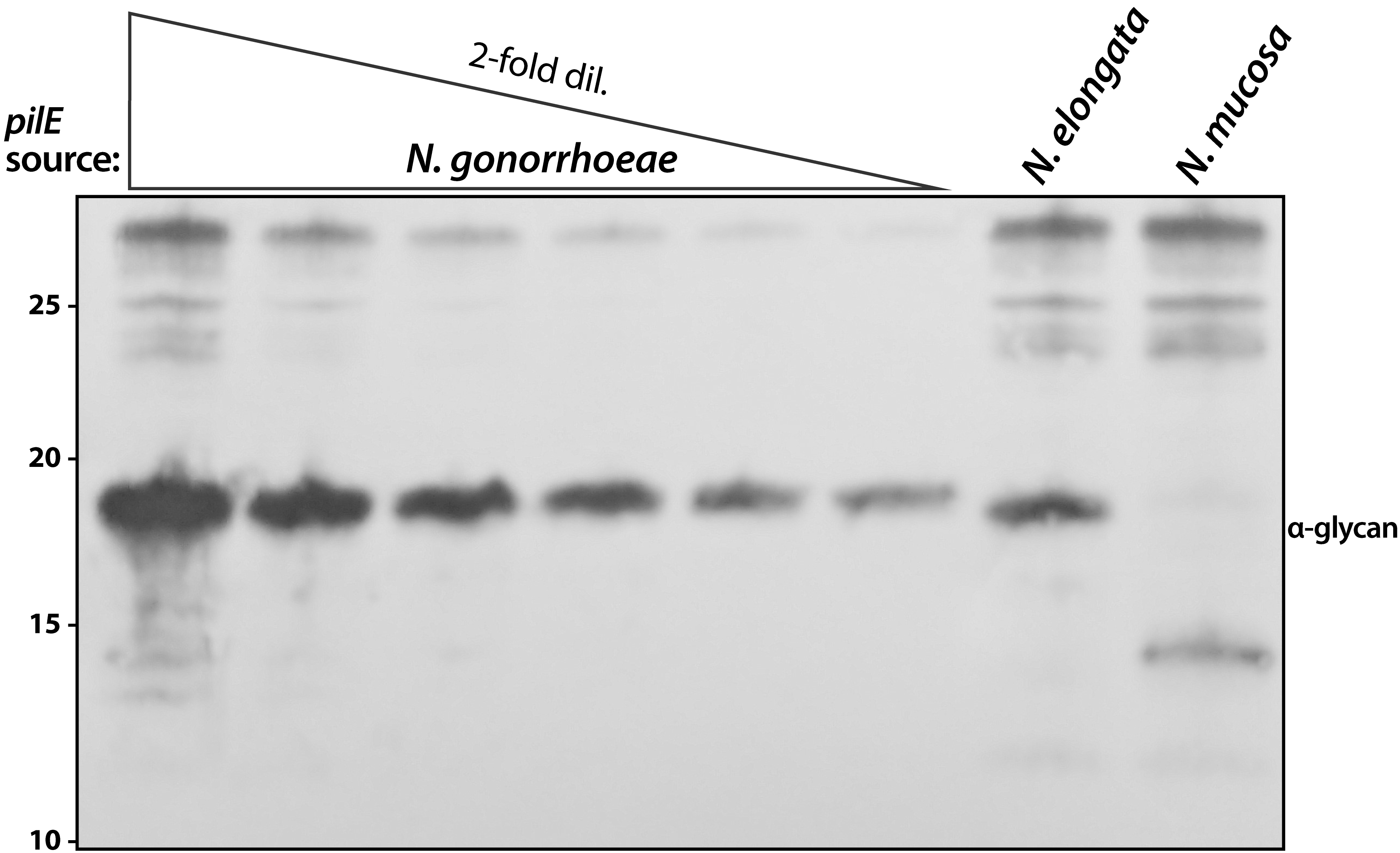

Supplement: FIG S2 [file mbio.03797-21-sf002.tif]

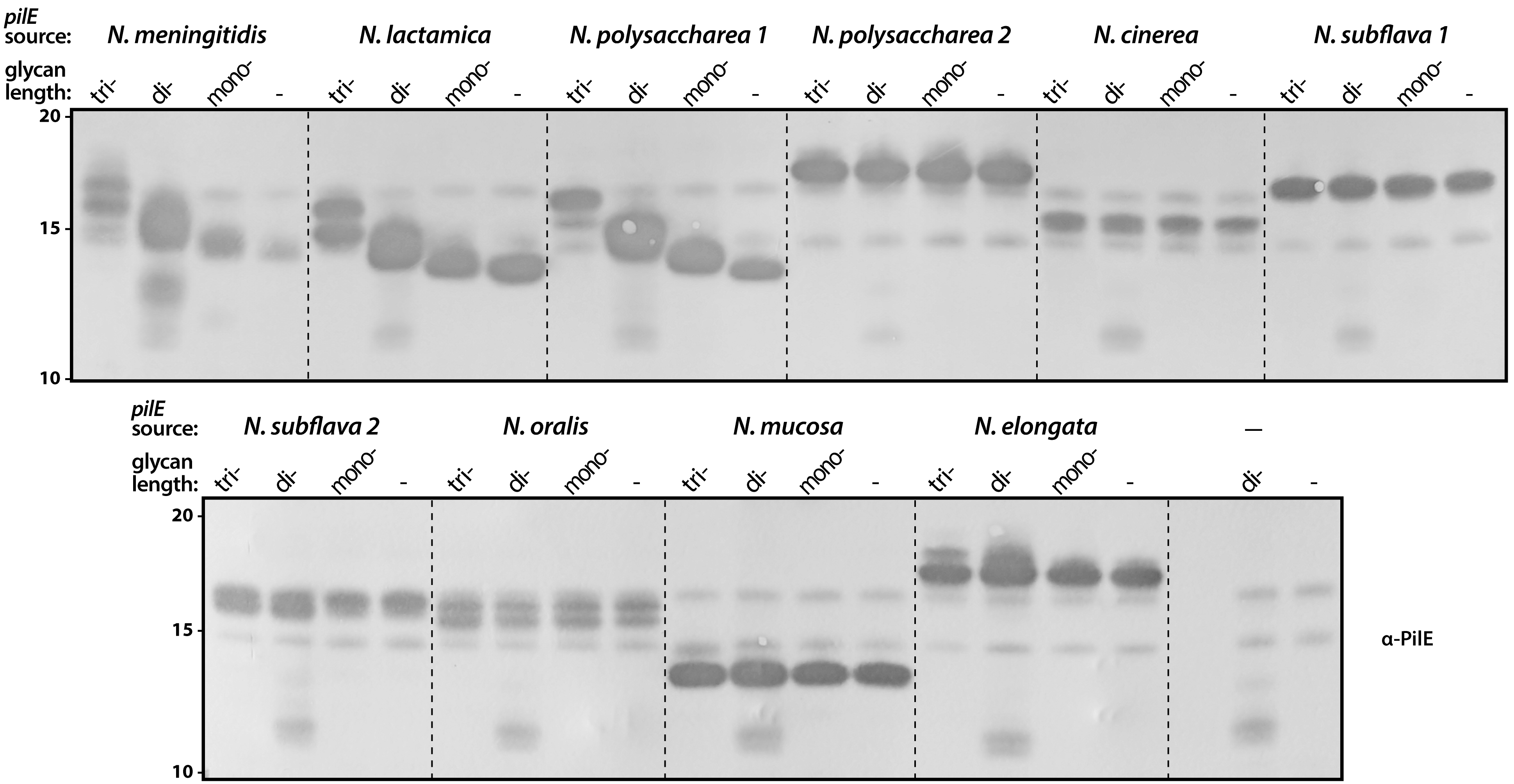

Supplement: FIG S4 [file mbio.03797-21-sf004.tif]

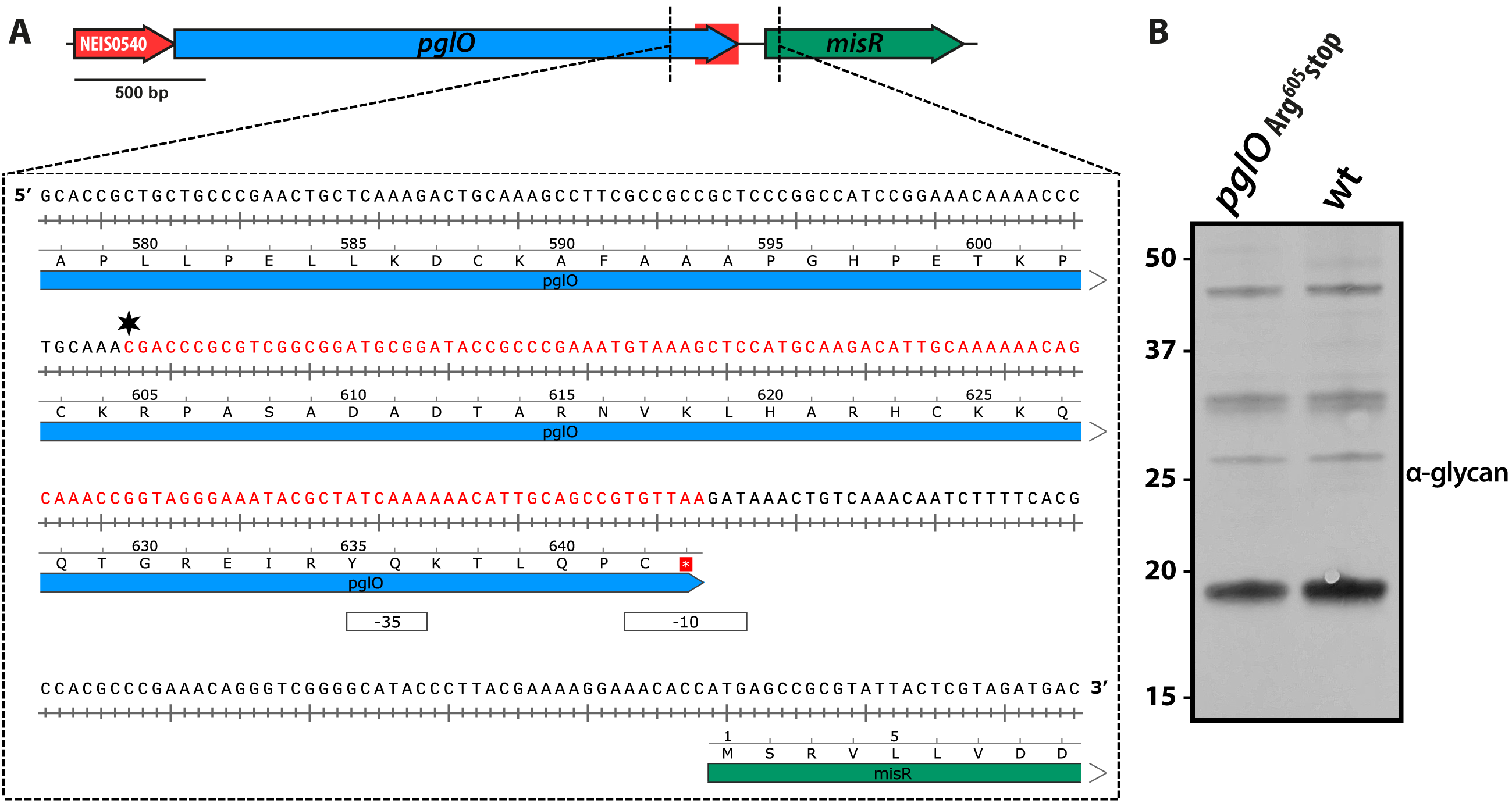

Supplement: FIG S5 [file mbio.03797-21-sf005.tif]

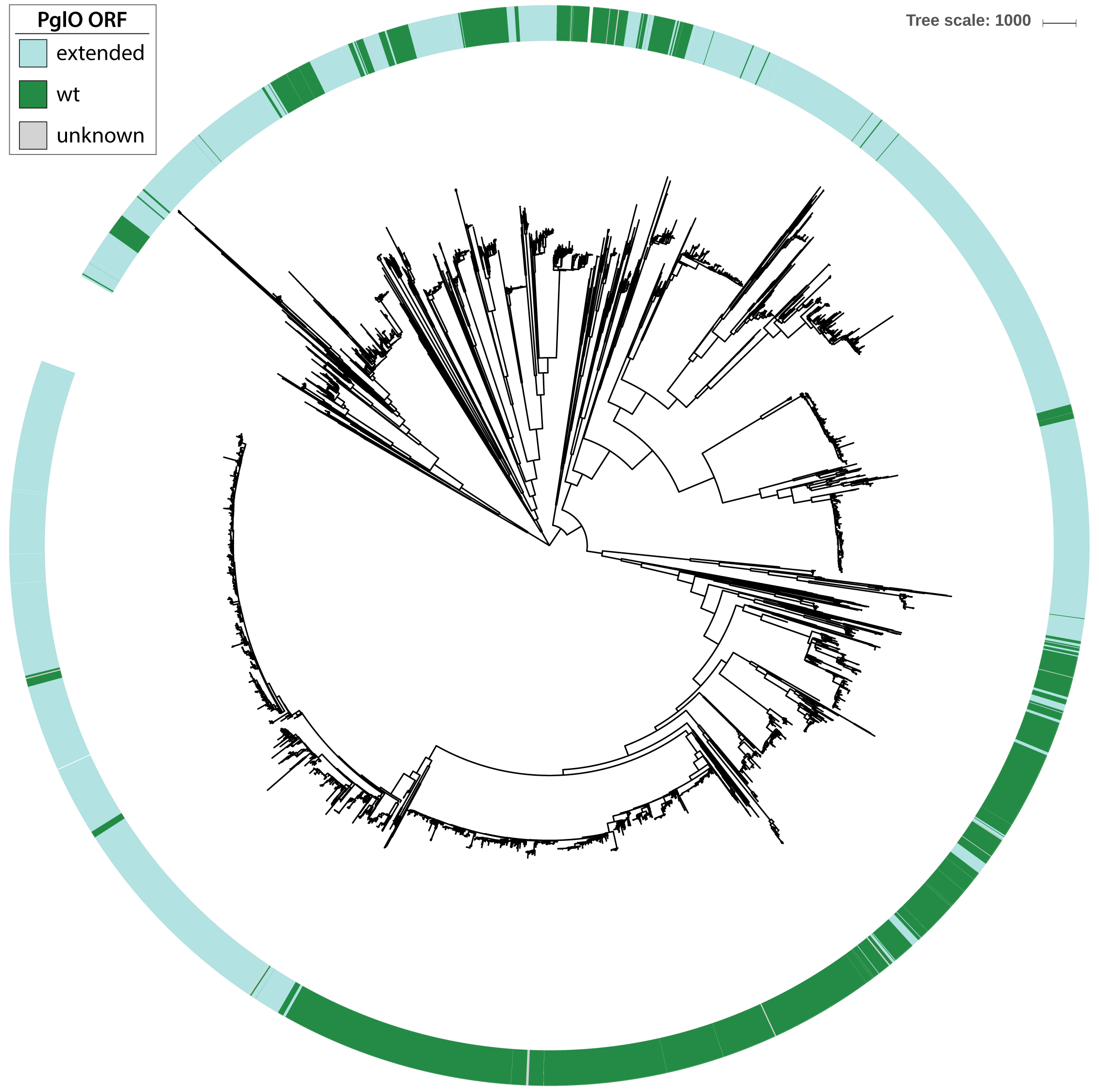

Supplement: FIG S6 [file mbio.03797-21-sf006.tif]

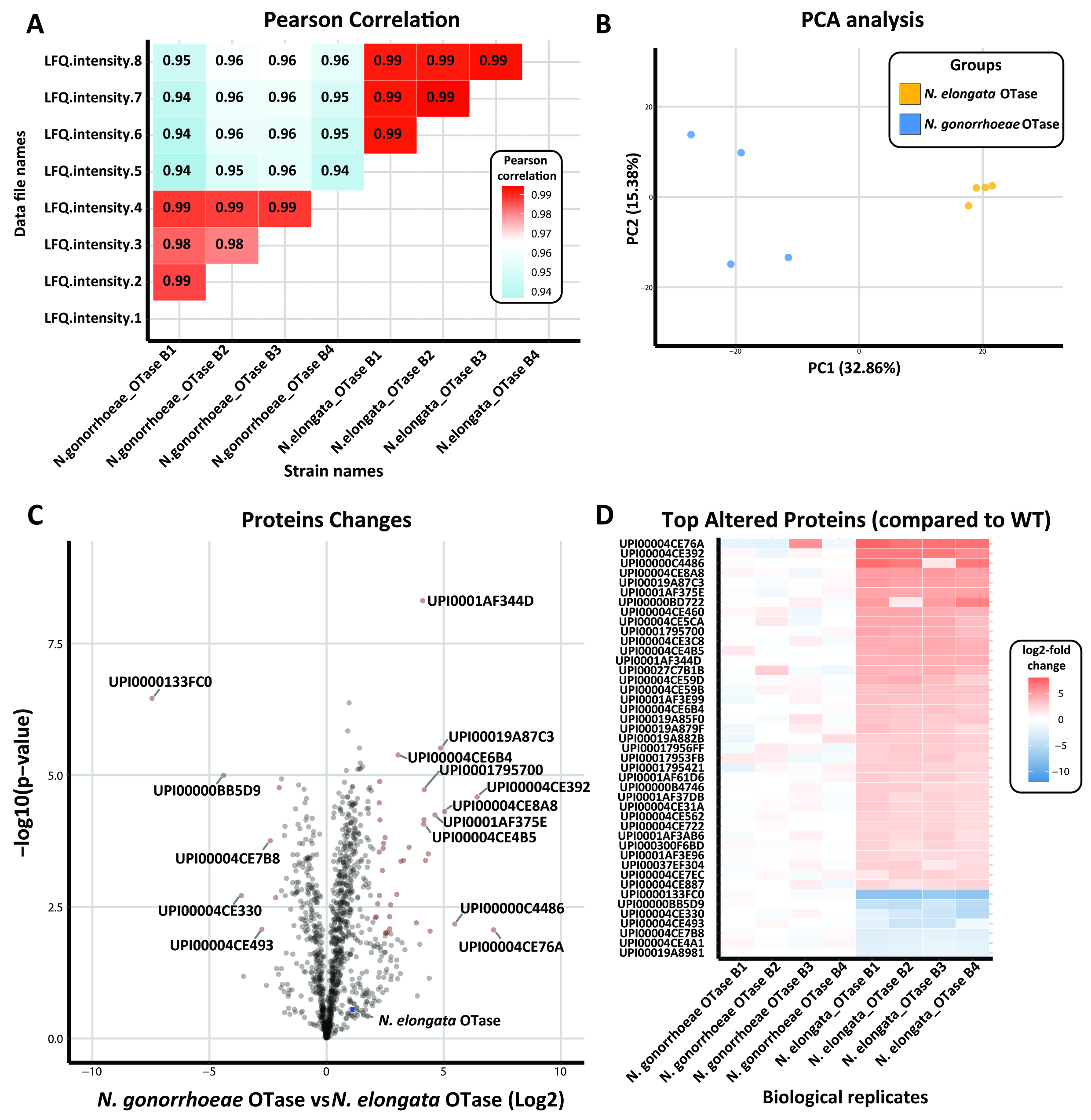

Supplement: FIG S7 [file mbio.03797-21-sf007.tif]

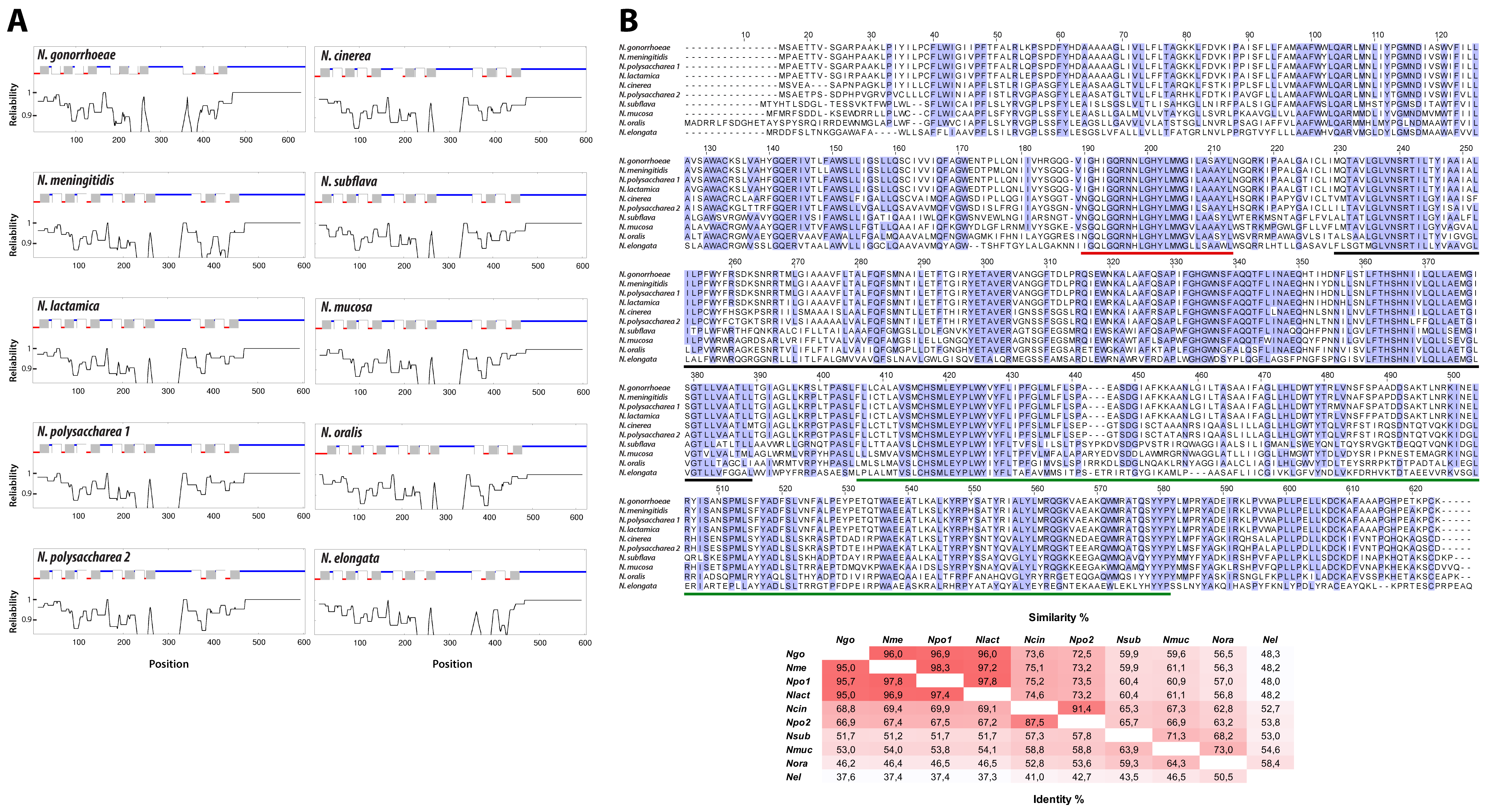

Supplement: FIG S8 [file mbio.03797-21-sf008.tif]

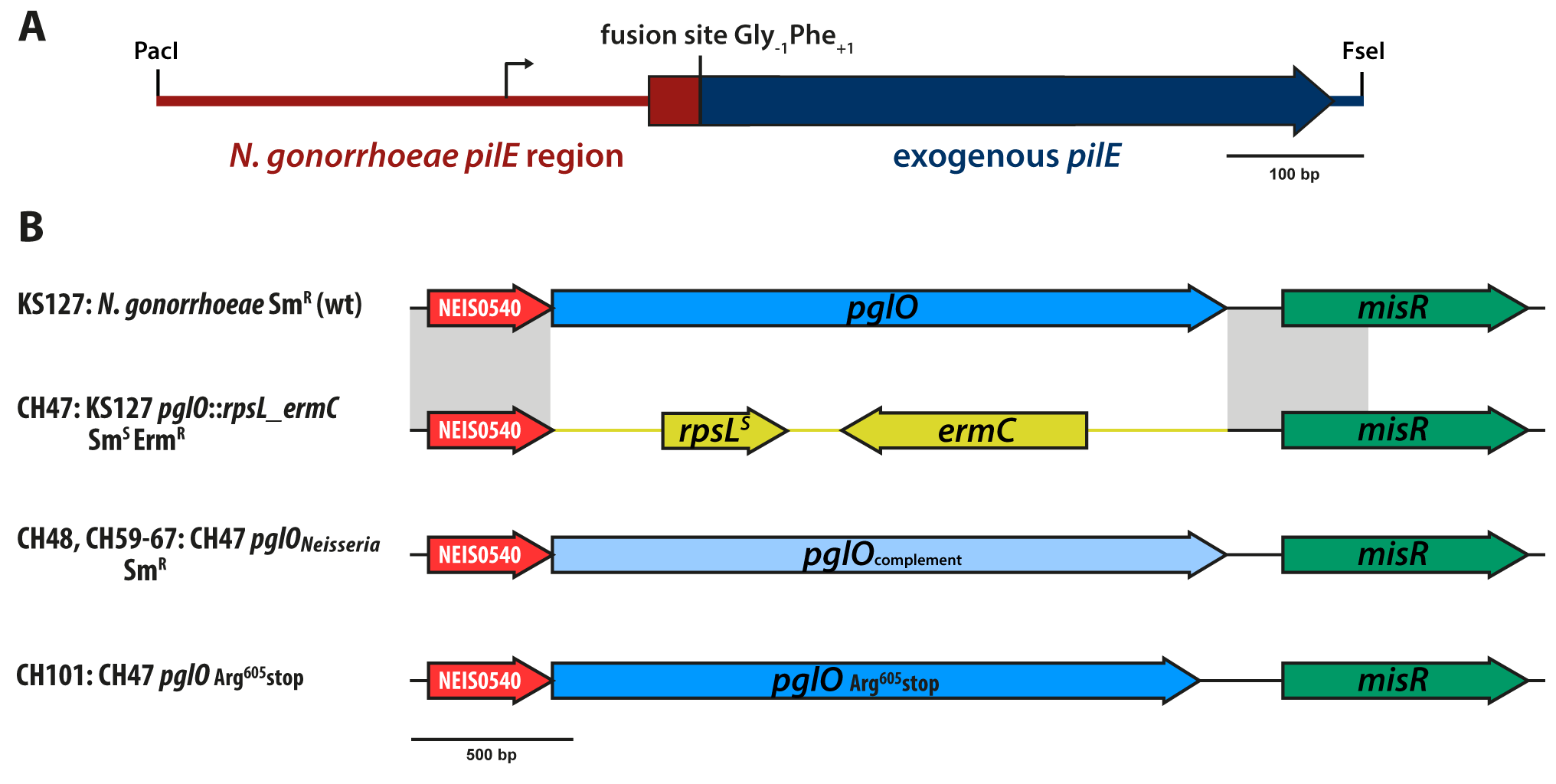

Supplement: FIG S9 [file mbio.03797-21-sf009.tif]
