# Supplementary material for: Sculpting the Bacterial O-Glycoproteome: Functional Analyses of Orthologous Oligosaccharyltransferases with Diverse Targeting Specificities
Source: mBio. 2022 Apr 26;13(3):e03797-21. doi: 10.1128/mbio.03797-21 (PMC9239064; doi:10.1128/mbio.03797-21)
Supplement: TABLE S1 [file mbio.03797-21-st001.docx]

| Table S1A. Strains and plasmids | |  |  |
| --- | --- | --- | --- |
| **Strain** | | **Genotype** | **Reference** |
|  | |  |  |
| *N. meningitidis* FAM18 | | - | Lab collection |
| *N. lactamica* ATCC 23970 | | - | ” |
| *N. polysaccharea* ATCC 43768 (*Npo*1) | | - | ” |
| *N. polysaccharea* CCUG 4790 (*Npo*2) | | - | ” |
| *N. cinerea* ATCC 14685 | | - | ” |
| *N. subflava* CCUG 23930 (*Nsub*1) | | - | ” |
| *N. subflava* CCUG 17913 (*Nsub*2) | | - | ” |
| *N. mucosa* CCUG 41451 | | - | ” |
| *N. oralis* CCUG 26878 | | - | ” |
| *N. elongata* subsp. *glycolytica* ATCC 29315 | | - | ” |
| KS101 — *N. gonorrhoeae* MS11 | | *pilE*_ind_::*tetM* | (5) |
| KS105 | | KS101 *pglC*::*kan* | (6) |
| KS122 | | KS101 *pglA*::*kan* | (7) |
| KS127 | | KS101 *pglE*_on_ | ” |
| CH114 | | KS122 *pilE _N. gonorrhoeae_* | This study |
| CH2 | | KS101 *pilE _N. elongata_* | ” |
| CH3 | | KS105 *pilE _N. elongata_* | ” |
| CH4 | | KS122 *pilE _N. elongata_* | ” |
| CH5 | | KS127 *pilE _N. elongata_* | ” |
| CH6 | | KS101 *pilE _N. cinerea_* | ” |
| CH7 | | KS105 *pilE _N. cinerea_* | ” |
| CH8 | | KS122 *pilE _N. cinerea_* | ” |
| CH9 | | KS127 *pilE _N. cinerea_* | ” |
| CH10 | | KS101 *pilE _N. polysaccharea_* _1_ | ” |
| CH11 | | KS105 *pilE _N. polysaccharea_* _1_ | ” |
| CH12 | | KS122 *pilE _N. polysaccharea_* _1_ | ” |
| CH13 | | KS127 *pilE _N. polysaccharea_* _1_ | ” |
| CH14 | | KS101 *pilE _N. lactamica_* | ” |
| CH15 | | KS105 *pilE _N. lactamica_* | ” |
| CH16 | | KS122 *pilE _N. lactamica_* | ” |
| CH17 | | KS127 *pilE _N. lactamica_* | ” |
| CH18 | | KS101 *pilE _N. subflava_* _1_ | ” |
| CH19 | | KS105 *pilE _N. subflava_* _1_ | ” |
| CH20 | | KS122 *pilE _N. subflava_* _1_ | ” |
| CH21 | | KS127 *pilE _N. subflava_* _1_ | ” |
| CH22 | | KS101 *pilE _N. meningitidis_* | ” |
| CH23 | | KS105 *pilE _N. meningitidis_* | ” |
| CH24 | | KS122 *pilE _N. meningitidis_* | ” |
| CH25 | | KS127 *pilE _N. meningitidis_* | ” |
| CH26 | | KS101 *pilE _N. subflava_* _2_ | ” |
| CH27 | | KS105 *pilE _N. subflava_* _2_ | ” |
| CH28 | | KS122 *pilE _N. subflava_* _2_ | ” |
| CH29 | | KS127 *pilE _N. subflava_* _2_ | ” |
| CH30 | | KS101 *pilE _N. oralis_* | ” |
| CH31 | | KS105 *pilE _N. oralis_* | ” |
| CH32 | | KS122 *pilE _N. oralis_* | ” |
| CH33 | | KS127 *pilE _N. oralis_* | ” |
| CH34 | | KS101 *pilE _N. mucosa_* | ” |
| CH35 | | KS105 *pilE _N. mucosa_* | ” |
| CH36 | | KS122 *pilE _N. mucosa_* | ” |
| CH37 | | KS127 *pilE _N. mucosa_* | ” |
| CH76 | | KS101 *pilE _N. polysaccharea 2_* | ” |
| CH77 | | KS105 *pilE _N. polysaccharea 2_* | ” |
| CH78 | | KS122 *pilE _N. polysaccharea 2_* | ” |
| CH79 | | KS127 *pilE _N. polysaccharea 2_* | ” |
| CH47 | | KS127 *pglO*::*rpsL_ermC* | ” |
| CH48 | | CH47 *pglO _N. elongata_* | ” |
| CH59 | | CH47 *pglO _N. cinerea_* | ” |
| CH60 | | CH47 *pglO _N. subflava_* _1_ | ” |
| CH61 | | CH47 *pglO _N. mucosa_* | ” |
| CH62 | | CH47 *pglO _N. oralis_* | ” |
| CH63 | | CH47 *pglO _N. polysaccharea_* _1_ | ” |
| CH64 | | CH47 *pglO _N. polysaccharea_* _2_ | ” |
| CH65 | | CH47 *pglO _N. lactamica_* | ” |
| CH66 | | CH47 *pglO _N. meningitidis_* | ” |
| CH91 | | CH47 *pglO* _hybrid 1_ | ” |
| CH92 | | CH47 *pglO* _hybrid 2_ | ” |
| CH93 | | CH47 *pglO* _hybrid 3_ | ” |
| CH94 | | CH47 *pglO* _hybrid 4_ | ” |
| CH95 | | CH47 *pglO* _hybrid 5_ | ” |
| CH96 | | CH47 *pglO* _hybrid 6_ | ” |
| CH97 | | CH47 *pglO* _hybrid 7_ | ” |
| CH98 | | CH47 *pglO* _hybrid 8_ | ” |
| CH99 | | CH47 *pglO* _hybrid 9_ | ” |
| CH100 | | CH47 *pglO* _hybrid 10_ | ” |
| CH105 | | CH47 *pglO* _hybrid 11_ | ” |
| CH106 | | CH47 *pglO* _hybrid 12_ | ” |
| CH152 | | CH47 *pglO* _hybrid 16_ | ” |
| CH101 | | CH47 *pglO* Arg^605^stop | ” |
| CH155 | | CH48 *laz*::*kan* | ” |
| CH156 | | CH48 *lip*::*kan* | ” |
| CH157 | | CH155 *lip*::*kan* | ” |
|  | |  |  |
| **Plasmid** | **Description** | | **Reference** |
|  |  | |  |
| pGCC6 | Contains a polylinker flanked by the *N. gonorrhoeae* genes *lctP*-*aspC* and used for ectopic expression | | (8) |
| pSY6 | Contains *gyrB* that confers resistance to nalidixic acid when transformed into *N. gonorrhoeae* | | (9) |
| pFLOB4300 | Contains a selectable (*ermC*) and a counter-selectable (*rpsL*) marker for creating marker-less mutations | | (10) |
| pCRII-TOPO | Vector used for creating plasmids | | Invitrogen |
| pKAN | Contains the kanamycin resistance cassette | | Lab collection |
| pCH59 | pCRII-TOPO vector carrying *N. cinerea* *pglO* with flanking homology to *N. gonorrhoeae pglO* | | This study |
| pCH66 | pCRII-TOPO vector carrying *N. meningitidis* *pglO* with flanking homology to *N. gonorrhoeae pglO* | | ” |
|  | |  |  |

Table S1B. PCR oligonucleotide primers

| **Primer** | **Sequence (5´ 3´)** | **Description** |
| --- | --- | --- |
|  |  |  |
|  | *pilE* constructs (Restriction sites) |  |
| CHP2 | gctcagggccggccggcctaatttgcctcatttgg | *Ngo* RVS |
| CHP4 | gctcagggccggcccgcatacagcggctctttc | *Nel* RVS |
| CHP7 | gcagcgttaattaaaattccgacccaatcaacacac | *Ngo* FWD |
| CHP10 | aaggcttcaccctgatcgagctgatg | *Npo*1 homology |
| CHP11 | catcagctcgatcagggtgaagcctt |  |
| CHP12 | agttaaggccggcccatttgttttcacggccgagtc | *Npo*1 RVS |
| CHP13 | caaaaaggctttaccctgatcga | *Nsub*2 homology |
| CHP14 | tcgatcagggtaaagcctttttg |  |
| CHP16 | atacaggccggccaaatgccgtctgaaacagcc | *Nci* RVS |
| CHP19 | agttggccggcctttcacggcgagttcaaacc | *Nla* RVS |
| CHP20 | caaaaaggcttctcccttatcga | *Nsub*1 homology |
| CHP21 | tcgataagggagaagcctttttg |  |
| CHP22 | actcaggccggccgctttgagtcgaatatcttttg | *Nsub*1 RVS |
| CHP27 | caaaaaggcttcaccctgatcga | *Nel*, *Nora*, *Npo*2, *Nmuc* and *Nme* homology |
| CHP28 | tcgatcagggtgaagcctttttg |  |
| CHP68 | gctggccggccaccaacgagatacgctctattc | *Nora* RVS |
| CHP69 | gtacggccggccaacccggtcattgtcctta | *Nme* RVS |
| CHP70 | actcaggccggcccataacgttttgtttggttatggg | *Nsub*2 RVS |
| CHP71 | actcaggccggcccagagcgagctttgttattcac | *Nmuc* RVS |
| CHP151 | agttaaggccggcccgattgcttcttcattaagtagc | *Npo*2 RVS |
|  |  |  |
| CHP80 | gccatcgacgaagcactc | *gyrB* from pSY6 |
| CHP81 | gcggccgtctgaaacgatt |  |
|  |  |  |
|  |  |  |
|  | *pglO* allelic exchange |  |
| CHP66 | atgcgtgatgatttttccctgaccagcggataacaatttcacacagg | pFLOB4300 |
| CHP67 | ctactgcgcttcaggtctcgggcacccagtcacgacgttgtaaaacg |  |
| CHP56 | tggcattgatgcgtgccaagagttc | *Ngo* homology |
| CHP60 | ggtcagggaaaaatcatcacgcatgtttgtttccttgtattgtttgacgaacg |  |
| CHP105 | atttcagacggctgcccgtttatgaaacc |  |
| CHP106 | tgtttgtttccttgtattgtttgacg |  |
| CHP107 | gataaactgtcaaacaatcttttcacgcc | *Nel* |
| CHP62 | atgcgtgatgatttttccctgaccaac |  |
| CHP63 | ctactgcgcttcaggtctcgggca |  |
| CHP108 | gtcaaacaatacaaggaaacaaacaatgtccgttgaagcatccgc | *Nci* |
| CHP109 | cgtgaaaagattgtttgacagtttatctcagtcgcaggattgtgctttc |  |
| CHP110 | gtcaaacaatacaaggaaacaaacaatgacctaccacacactttcagacg | *Nsub*1 |
| CHP111 | cgtgaaaagattgtttgacagtttatcttagggtttgtcacatgatttggcag |  |
| CHP112 | gtcaaacaatacaaggaaacaaacaatgtttatgcgtttttcagacgaccttaag | *Nmuc* |
| CHP113 | cgtgaaaagattgtttgacagtttatcttattgcacaacatcgcaagacttcgc |  |
| CHP114 | gtcaaacaatacaaggaaacaaacaatggctgaccgccgctt | *Nora* |
| CHP115 | cgtgaaaagattgtttgacagtttatcctatttcggggcttcgcag |  |
| CHP152 | gtcaaacaatacaaggaaacaaacaatgcccgc | *Nme*, *Nla* and *Npo*1 |
| CHP153 | cgtgaaaagattgtttgacagtttatcttaacacggctgcaatg |  |
| CHP154 | gtcaaacaatacaaggaaacaaacaatgtccgctgaaacac | *Npo*2 (+CHP109) |
|  |  |  |
|  |  |  |
|  | *Ngo* mutagenesis |  |
| CHP215  CHP216  CHP217  CHP218 | ccactttcagacggcatgtatcga | *laz* upstream |
|  | ttttgagacacaacgtggcccagataagctttcatggt |  |
|  | caacaccttcttcacgaggagtgactttggtcgattaa | *laz* downstream |
|  | acggcattatttgcaccggc |  |
| CHP219  CHP220  CHP221  CHP222 | caggaacagcagattgattacaggcg | *lip* upstream |
|  | gattttgagacacaacgtggcgacaacaaagcagcggca |  |
|  | gcaacaccttcttcacgaggctgccgaagctgcaaaataag | *lip* downstream |
|  | tggagtacggaacctgcttcgac |  |
| CHP172 | gccacgttgtgtctcaaaatctctg | pKAN |
| CHP173 | cctcgtgaagaaggtgttgctg |  |
| CHP225 | gtcaagtcagcgtaatgctctgc |  |
| CHP212 | acaaaaccctgcaaatgacccgcgtcg | Arg^605^stop |
| CHP213 | ggtcatttgcagggttttgtttccg |  |
|  |  |  |
|  |  |  |
|  | *pglO* hybrids |  |
| CHP196 | ggtgcgcgaattgaccaggcct | hybrid 1 |
| CHP197 | tggtcaattcgcgcaccatct |  |
| CHP198 | tggtcaattcgcgcaccatcttaggc | hybrid 2 |
| CHP199 | atggtgcgcgaattgaccaaacct |  |
| CHP200 | cagggtaacgatgcgttcctgtc | hybrid 3 |
| CHP201 | gaacgcatcgttaccctgcttgcc |  |
| CHP202 | gaacgcatcgttaccctgtttgcctgg | hybrid 4 |
| CHP203 | cagggtaacgatgcgttcttgtccg |  |
| CHP204 | cgttcgacggcagtttcatagcg | hybrid 5 |
| CHP205 | gaaactgccgtcgaacgcgttg |  |
| CHP206 | gaaactgccgtcgaacgcatcg | hybrid 6 |
| CHP207 | cgttcgacggcagtttcgtagcg |  |
| CHP208 | acatcaggtagtgtccgagatggttgc | hybrid 7 |
| CHP209 | ctcggacactacctgatgtgggg |  |
| CHP210 | gacactacctgatgtgggggatactagcc | hybrid 8 |
| CHP211 | ccccacatcaggtagtgtccgag |  |
| CHP167 | ctccaactccttgcagagatgg | hybrid 9 |
| CHP168 | ctctgcaaggagttggagaatgatgttg |  |
| CHP169 | tctgcaaggagttggaggacgatg | hybrid 10 |
| CHP170 | cctccaactccttgcagaaaccg |  |
| CHP223 | gggtgtatgtccagtccaagtgca | hybrid 11 |
| CHP224 | ttggactggacatacacccaactggttcg |  |
| CHP165 | ttaggctggacatacacccggctg | hybrid 12 |
| CHP166 | gggtgtatgtccagcctaagtgc |  |
|  |  |  |
|  |  |  |

Table S1C. List of isolates used for genus-wide phylogenetic analyses in *Neisseria*

|  | **PubMLST ID** | **Isolate ID** | **Country** | **Year** | **Serogroup** | **ST (MLST)** |
| --- | --- | --- | --- | --- | --- | --- |
| ***N. gonorrhoeae*** |  |  |  |  |  |  |
|  | 2855 | FA1090 | Unknown |  |  | 1899 |
|  | 13685 | NCCP11945 | South Korea |  |  | 1901 |
|  | 15698 | DGI2 | USA |  |  | 8421 |
|  | 15935 | PID24 | USA |  |  | 8418 |
|  | 15936 | DGI18 | USA |  |  | 8418 |
|  | 21065 | TCDC-NG08107 | Unknown | 2008 |  | 7363 |
|  | 21066 | PID18 | Unknown |  |  | 1926 |
|  | 21067 | 1291 | Unknown |  |  | 8422 |
|  | 21068 | PID1 | Unknown |  |  | 10154 |
|  | 21070 | F62 | Unknown |  |  | 1900 |
|  | 21071 | SK-92-679 | Unknown |  |  | 6715 |
|  | 21072 | SK-93-1035 | Unknown |  |  | 1595 |
|  | 21073 | PID332 | Unknown |  |  | 1594 |
|  | 46275 | FA19 | Unknown |  |  | 1892 |
|  | 46276 | FA6140 | USA |  |  | 1927 |
|  | 46278 | SRR515980 | Unknown |  |  | 6959 |
|  |  |  |  |  |  |  |
| ***N. meningitidis*** |  |  |  |  |  |  |
|  | 1 | A4/M1027 | USA | 1937 | A | 4 |
|  | 2 | 120M | Pakistan | 1967 | A | 1 |
|  | 7 | 7891 | Finland | 1975 | A | 5 |
|  | 10 | 6748 | Canada | 1971 | A | 1 |
|  | 11 | 129E | Germany | 1964 | A | 1 |
|  | 13 | 139M | Philippines | 1968 | A | 1 |
|  | 19 | S3131 | Ghana | 1973 | A | 4 |
|  | 24 | S4355 | Denmark | 1974 | A | 5 |
|  | 31 | 10 | Burkina Faso | 1963 | A | 4 |
|  | 34 | 20 | Niger | 1963 | A | 1 |
|  | 35 | 26 | Niger | 1963 | A | 4 |
|  | 52 | 243 | Cameroon | 1966 | A | 4 |
|  | 61 | 393 | Greece | 1968 | A | 1 |
|  | 64 | 254 | Djibouti | 1966 | A | 1 |
|  | 67 | S5611 | Australia | 1977 | A | 1 |
|  | 82 | 11-004 | China | 1984 | A | 5 |
|  | 84 | IAL2229 | Brazil | 1976 | A | 5 |
|  | 90 | CN100 | UK | 1941 | A | 21 |
|  | 120 | F4698 | Saudi Arabia | 1987 | A | 5 |
|  | 128 | F6124 | Chad | 1988 | A | 5 |
|  | 160 | 1014 | Sudan | 1985 | A | 4 |
|  | 210 | H1964 | UK | 1987 | A | 5 |
|  | 237 | H44/76 | Norway | 1976 | B | 32 |
|  | 238 | 153 | China | 1966 | A | 5 |
|  | 239 | 154 | China | 1966 | A | 6 |
|  | 299 | 80049 | China | 1963 | A | 5 |
|  | 314 | D1 | Mali | 1989 | C | 11 |
|  | 316 | D8 | Mali | 1990 | A | 4 |
|  | 340 | 196/87 | Norway | 1987 | C | 32 |
|  | 343 | 500 | Italy | 1984 | C | 11 |
|  | 349 | 38VI | USA | 1964 | B | 11 |
|  | 369 | M597 | Israel | 1988 | C | 11 |
|  | 387 | 2059001 | Mali | 1990 | A | 4 |
|  | 391 | 90/18311 | UK | 1990 | C | 11 |
|  | 398 | BZ 10 | Netherlands | 1967 | B | 8 |
|  | 400 | BZ 83 | Netherlands | 1984 | B | 34 |
|  | 403 | BZ 147 | Netherlands | 1963 | B | 48 |
|  | 407 | BZ 163 | Netherlands | 1979 | B | 9 |
|  | 408 | BZ 169 | Netherlands | 1985 | B | 32 |
|  | 409 | BZ 198 | Netherlands | 1986 | B | 41 |
|  | 410 | BZ 232 | Netherlands | 1964 | B | 38 |
|  | 411 | DK 24 | Denmark | 1940 | B | 16 |
|  | 412 | DK 353 | Denmark | 1962 | B | 37 |
|  | 414 | EG 327 | Germany | 1985 | B | 19 |
|  | 415 | EG 329 | Germany | 1985 | B | 32 |
|  | 416 | EG 011 | Germany | 1986 | B | 36 |
|  | 417 | NG 3/88 | Norway | 1988 | B | 12 |
|  | 418 | NG 4/88 | Norway | 1988 | B | 30 |
|  | 419 | NG 6/88 | Norway | 1988 | B | 13 |
|  | 420 | NG F26 | Norway | 1988 | B | 13 |
|  | 421 | NG H15 | Norway | 1988 | B | 43 |
|  | 423 | NG H38 | Norway | 1988 | B | 36 |
|  | 424 | NG E31 | Norway | 1988 | B | 15 |
|  | 425 | NG G40 | Norway | 1988 | B | 25 |
|  | 427 | NG E30 | Norway | 1988 | B | 44 |
|  | 428 | NG H36 | Norway | 1988 | B | 47 |
|  | 430 | NG 080 | Norway | 1981 | B | 32 |
|  | 431 | NG144/82 | Norway | 1982 | B | 32 |
|  | 434 | NG PB24 | Norway | 1985 | B | 32 |
|  | 441 | 8680 | Chile | 1987 | B | 32 |
|  | 442 | 297-0 | Chile | 1987 | B | 8523 |
|  | 443 | 3906 | China | 1977 | B | 17 |
|  | 445 | 528 | Russia | 1989 | B | 18 |
|  | 446 | 1000 | Russia | 1988 | B | 20 |
|  | 451 | 14/1455 | Russia | 1970 | A | 5 |
|  | 466 | 371 | India | 1980 | A | 1 |
|  | 467 | 690 | India | 1980 | A | 4 |
|  | 468 | BRAZ10 | Brazil | 1976 | C | 11 |
|  | 488 | 106 | Morocco | 1967 | A | 1 |
|  | 492 | 79128 | China | 1979 | A | 3 |
|  | 493 | 322/85 | Germany | 1985 | A | 2 |
|  | 494 | 79126 | China | 1979 | A | 3 |
|  | 507 | MA-5756 | Spain | 1985 | C | 11 |
|  | 597 | 92001 | China | 1992 | A | 7 |
|  | 613 | Z2491 | The Gambia | 1983 | A | 4 |
|  | 638 | G2136 | UK | 1986 | B | 8 |
|  | 640 | SB25 | South Africa | 1990 | C | 8 |
|  | 641 | 94/155 | New Zealand | 1994 | C | 66 |
|  | 642 | 312 901 | UK | 1996 | C | 8 |
|  | 643 | AK22 | Greece | 1992 | B | 153 |
|  | 644 | L93/4286 | UK | 1993 | C | 11 |
|  | 645 | 204/92 | Cuba | 1992 | B | 33 |
|  | 646 | 400 | Austria | 1991 | B | 40 |
|  | 647 | AK50 | Greece | 1992 | B | 41 |
|  | 648 | M-101/93 | Iceland | 1993 | B | 41 |
|  | 649 | 50/94 | Norway | 1994 | B | 45 |
|  | 650 | M40/94 | Chile | 1994 | B | 41 |
|  | 651 | 931905 | Netherlands | 1993 | B | 41 |
|  | 652 | N45/96 | Norway | 1996 | B | 41 |
|  | 653 | 91/40 | New Zealand | 1991 | B | 42 |
|  | 655 | E32 | Norway | 1988 | Z | 31 |
|  | 656 | E26 | Norway | 1988 | X | 39 |
|  | 657 | 860060 | Netherlands | 1986 | X | 24 |
|  | 659 | A22 | Norway | 1986 | W | 22 |
|  | 660 | 71/94 | Norway | 1994 | Y | 23 |
|  | 661 | 860800 | Netherlands | 1986 | Y | 29 |
|  | 19363 | 961-5945 | Unknown |  |  | 153 |
|  | 34542 | NZ98/254 | New Zealand | 1998 | B | 42 |
|  | 35956 | 255 | Burkina Faso | 1966 | A | 4 |
|  | 35957 | 890326 | Netherlands | 1989 | Z | 28 |
|  | 35958 | BZ 133 | Netherlands | 1977 | B | 10300 |
|  | 35959 | EG 328 | Germany | 1985 | B | 18 |
|  | 35960 | F1576 | Ghana | 1984 | C | 11 |
|  | 35961 | NG E28 | Norway | 1988 | B | 26 |
|  | 35962 | NG H41 | Norway | 1988 | B | 27 |
|  | 35963 | NG P20 | Norway | 1969 | B | 11 |
|  | 35964 | SWZ107 | Switzerland | 1986 | B | 35 |
|  | 38305 | 88/03415 | UK | 1988 | B | 46 |
|  | 47014 | B6116/77 | Iceland | 1977 | B | 10 |
|  |  |  |  |  |  |  |
| ***N. polysaccharea*** |  |  |  |  |  |  |
|  | 14730 | ATCC 43768 | Belgium |  |  | 3557 |
|  | 21047 | CCUG 4790 | Unknown |  |  | 10257 |
|  | 36140 | 12015_2014 | Ireland | 2014 | other | 11374 |
|  | 36142 | 12017_2014 | Ireland | 2014 | NG | 2985 |
|  | 36148 | 12024_2014 | Ireland | 2014 | other | 11376 |
|  | 36153 | 12030_2014 | Ireland | 2014 | other | 11378 |
|  | 36167 | 12046_2014 | Ireland | 2014 | other | 11382 |
|  | 41474 | M15 240880 | UK | 2015 | NG | 3574 |
|  | 41528 | M15 240955 | UK | 2015 | NG | 3574 |
|  | 41652 | 2748 | Italy | 2015 |  | 11879 |
|  | 42468 | M16 240183 | UK | 2016 | NG | 3574 |
|  | 42472 | M15 240827 | UK | 2015 | NG | 11879 |
|  | 44837 | M16 240285 | UK | 2016 | NG |  |
|  | 49356 | CCUG 18031 | Germany | 1984 |  | 3557 |
|  | 49357 | CCUG 24845 | Unknown |  |  | 3581 |
|  | 49358 | CCUG 24846 | Unknown |  |  | 3574 |
|  | 49359 | CCUG 27182 | Unknown |  |  | 9811 |
|  | 53400 | M17 240155 | Unknown |  |  |  |
|  |  |  |  |  |  |  |
| ***N. lactamica*** |  |  |  |  |  |  |
|  | 1770 | 028-12 | UK | 1997 |  | 584 |
|  | 1777 | 049-12 | UK | 1998 |  | 591 |
|  | 1780 | 016-24 | UK | 1998 |  | 594 |
|  | 1809 | 224 | UK | 2000 |  | 624 |
|  | 1827 | 005-12 | UK | 1997 |  | 642 |
|  | 4410 | OX9931639 | UK | 1999 | NG | 3260 |
|  | 4945 | Y92-1009 | UK | 1992 |  | 3493 |
|  | 5544 | ATCC 23970 | USA | 1969 |  | 3787 |
|  | 8778 | 004-12 | UK | 1997 |  | 595 |
|  | 8790 | 012-12 | UK | 1997 |  | 631 |
|  | 8837 | 017-02 | UK | 1997 |  | 608 |
|  | 8851 | 020-06 | UK | 1997 |  | 640 |
|  | 8917 | 039-03 | UK | 1997 |  | 582 |
|  | 26478 | BB97 | UK | 2012 |  | 3493 |
|  | 27000 | 90142v4 | UK |  |  | 613 |
|  | 29272 | BM35b | Greece | 1996 | NG | 1209 |
|  | 29274 | BM68a | Greece | 1997 | NG | 11143 |
|  | 30770 | M45.1 | Ireland | 2013 | B | 10984 |
|  | 30771 | M46.2 | Ireland | 2013 |  | 10984 |
|  | 36172 | M10.1 | Ireland | 2013 |  | 1494 |
|  | 36173 | M11.1 | Ireland | 2013 |  | 1494 |
|  | 36174 | M15.1 | Ireland | 2013 |  | 11383 |
|  | 36175 | M17.1 | Ireland | 2013 |  | 10984 |
|  | 37261 | 07AZI_T_009 | USA |  |  | 595 |
|  | 38175 | M37084 | Brazil | 2014 |  | 11457 |
|  | 42495 | M16 240028 | UK | 2016 | NG | 12018 |
|  | 46077 | M46.10 | Ireland | 2013 |  |  |
|  | 46267 | NS19 | Unknown |  |  | 12442 |
|  | 49335 | 030-24 | UK | 1999 |  | 585 |
|  | 49336 | 014-24 | UK | 1998 |  | 586 |
|  | 49337 | 09002S1 | UK | 1999 |  | 601 |
|  | 49338 | 8206 | UK | 2000 |  | 604 |
|  |  |  |  |  |  |  |
| ***N. cinerea*** |  |  |  |  |  |  |
|  | 14731 | ATCC 14685 | Unknown |  |  | 3579 |
|  | 21041 | CCUG 53043 | Sweden | 2006 |  | 10252 |
|  | 26871 | 12008_2012 | Ireland | 2012 | other |  |
|  | 26876 | 12013_2012 | Ireland | 2012 | other |  |
|  | 36161 | 12040_2014 | Ireland | 2014 | other | 11381 |
|  | 42462 | M16 240153 | UK | 2016 | NG |  |
|  | 42471 | M15 240769 | UK | 2015 | NG | 11878 |
|  | 42503 | M16 240038 | UK | 2016 | NG |  |
|  | 47345 | M16 240660 | UK | 2016 |  | 12552 |
|  | 49341 | CCUG 346 T | Unknown |  |  | 9331 |
|  | 49342 | CCUG 5746 | Sweden | 1977 |  | 9332 |
|  | 49343 | CCUG 25879 | Sweden | 1989 |  | 9333 |
|  | 49344 | CCUG 27178 A | Unknown | 1983 |  | 9334 |
|  | 49350 | CCUG 28662 | Sweden | 1991 |  | 9338 |
|  | 50859 | 16-530 | Sweden | 2016 |  | 12552 |
|  |  |  |  |  |  |  |
| ***N. subflava*** |  |  |  |  |  |  |
|  | 14732 | NRL30031 | Unknown |  |  | 3576 |
|  | 14733 | NJ9703 | Unknown |  |  | 9805 |
|  | 21060 | C102 | Unknown |  |  | 10149 |
|  | 21063 | CCUG 24841 | Unknown |  |  | 10152 |
|  | 21064 | CCUG 24844 | Unknown |  |  | 10153 |
|  | 26870 | 12007_2012 | Ireland | 2012 | other | 12151 |
|  | 46032 | 12026-15 | Ireland | 2015 | E | 11098 |
|  | 46033 | 12027-15A | Ireland | 2015 |  | 13245 |
|  | 46034 | 12029-15A | Ireland | 2015 |  | 13245 |
|  | 46079 | T-20455-10 | Ireland | 2015 |  |  |
|  | 46268 | 583_NLAC | USA |  |  |  |
|  | 46269 | 595_NLAC | USA |  |  |  |
|  | 46753 | C6A | Malaysia | 2012 |  |  |
|  | 49346 | CCUG 4788 | Unknown |  |  | 9336 |
|  | 49347 | CCUG 29761 | Sweden | 1992 |  | 9337 |
|  | 49348 | CCUG 17913 T | USA |  |  | 3576 |
|  | 49349 | CCUG 806 | Unknown |  |  | 3576 |
|  | 49353 | CCUG 7826 | Sweden | 1979 |  | 10174 |
|  | 49362 | CCUG 24918 | Sweden | 1989 |  | 9342 |
|  | 49363 | CCUG 23930 T | Unknown |  |  | 9344 |
|  | 49364 | CCUG 800 | Unknown |  |  | 9343 |
|  | 49365 | CCUG 801 | Unknown |  |  | 9343 |
|  | 49366 | CCUG 24960 | Unknown |  |  | 9344 |
|  | 49367 | CCUG 25198 | Sweden | 1989 |  | 9345 |
|  | 49373 | SK114 | Unknown |  |  | 10303 |
|  |  |  |  |  |  |  |
| ***N. mucosa*** |  |  |  |  |  |  |
|  | 2863 | ATCC 29256 | Unknown |  |  | 8081 |
|  | 3565 | ATCC 25996 | Unknown |  |  | 8082 |
|  | 5197 | ATCC 9913 | USA | 1952 |  | 3562 |
|  | 5204 | ATCC 19243 | USA | 1973 |  | 3569 |
|  | 5354 | ATCC 19696 | France | 1959 |  | 3706 |
|  | 21043 | CCUG 26474 | UK |  |  | 10254 |
|  | 21061 | CCUG 17327 | Sweden | 1985 |  | 10150 |
|  | 26577 | GT4A_CT1 | Unknown |  |  | 10462 |
|  | 27632 | CCUG 805 | Unknown |  |  | 8082 |
|  | 37778 | M14 240642 | UK | 2014 | NG |  |
|  | 46270 | 919_NLAC | USA |  |  |  |
|  | 49351 | CCUG 41451 T | Unknown |  |  | 9339 |
|  | 49352 | CCUG 12106 | Sweden | 1982 |  |  |
|  | 49354 | CCUG 32036 | Sweden | 1993 |  | 12047 |
|  | 49355 | CCUG 32112 | Sweden | 1993 |  | 12048 |
|  | 49360 | CCUG 431 | Unknown |  |  | 12049 |
|  | 49361 | CCUG 24847 | Unknown |  |  |  |
|  |  |  |  |  |  |  |
| ***N. oralis*** |  |  |  |  |  |  |
|  | 19091 | CCUG 26878 T | Unknown |  |  |  |
|  | 21044 | F0314 | USA | 1982 |  | 10248 |
|  | 21045 | CCUG 10421 | Sweden | 1980 |  | 10255 |
|  | 21046 | CCUG 804 | Unknown |  |  | 10256 |
|  |  |  |  |  |  |  |
| ***N. elongata*** |  |  |  |  |  |  |
|  | 14740 | ATCC 29315 | Unknown |  |  | 9806 |
|  | 20515 | CCUG 30802T | USA |  |  | 9905 |
|  | 20516 | CCUG 2043T | Unknown |  |  | 9906 |
|  |  |  |  |  |  |  |
|  |  |  |  |  |  |  |

**References**

1. Kumar S, Stecher G, Tamura K. 2016. MEGA7: Molecular Evolutionary Genetics Analysis Version 7.0 for Bigger Datasets. Mol Biol Evol 33:1870-4.

2. Letunic I, Bork P. 2016. Interactive tree of life (iTOL) v3: an online tool for the display and annotation of phylogenetic and other trees. Nucleic Acids Res 44:W242-5.

3. Ma KC, Mortimer TD, Duckett MA, Hicks AL, Wheeler NE, Sanchez-Buso L, Grad YH. 2020. Increased power from conditional bacterial genome-wide association identifies macrolide resistance mutations in *Neisseria gonorrhoeae*. Nat Commun 11:5374.

4. Tsirigos KD, Peters C, Shu N, Kall L, Elofsson A. 2015. The TOPCONS web server for consensus prediction of membrane protein topology and signal peptides. Nucleic Acids Res 43:W401-7.

5. Wolfgang M, van Putten JP, Hayes SF, Dorward D, Koomey M. 2000. Components and dynamics of fiber formation define a ubiquitous biogenesis pathway for bacterial pili. EMBO J 19:6408-18.

6. Hegge FT, Hitchen PG, Aas FE, Kristiansen H, Lovold C, Egge-Jacobsen W, Panico M, Leong WY, Bull V, Virji M, Morris HR, Dell A, Koomey M. 2004. Unique modifications with phosphocholine and phosphoethanolamine define alternate antigenic forms of *Neisseria gonorrhoeae* type IV pili. Proc Natl Acad Sci U S A 101:10798-803.

7. Aas FE, Winther-Larsen HC, Wolfgang M, Frye S, Lovold C, Roos N, van Putten JP, Koomey M. 2007. Substitutions in the N-terminal alpha helical spine of *Neisseria gonorrhoeae* pilin affect Type IV pilus assembly, dynamics and associated functions. Mol Microbiol 63:69-85.

8. Mehr IJ, Long CD, Serkin CD, Seifert HS. 2000. A homologue of the recombination-dependent growth gene, *rdgC*, is involved in gonococcal pilin antigenic variation. Genetics 154:523-32.

9. Stein DC, Danaher RJ, Cook TM. 1991. Characterization of a *gyrB* mutation responsible for low-level nalidixic acid resistance in *Neisseria gonorrhoeae*. Antimicrob Agents Chemother 35:622-6.

10. Johnston DM, Cannon JG. 1999. Construction of mutant strains of *Neisseria gonorrhoeae* lacking new antibiotic resistance markers using a two gene cassette with positive and negative selection. Gene 236:179-84.
